# A Kinome-Wide RNAi Screen in *Drosophila* Glia Reveals That the RIO Kinases Mediate Cell Proliferation and Survival through TORC2-Akt Signaling in Glioblastoma

**DOI:** 10.1371/journal.pgen.1003253

**Published:** 2013-02-14

**Authors:** Renee D. Read, Tim R. Fenton, German G. Gomez, Jill Wykosky, Scott R. Vandenberg, Ivan Babic, Akio Iwanami, Huijun Yang, Webster K. Cavenee, Paul S. Mischel, Frank B. Furnari, John B. Thomas

**Affiliations:** 1 Molecular Neurobiology Laboratory, Salk Institute for Biological Studies, La Jolla, California, United States of America; 2 Ludwig Institute for Cancer Research, University of California San Diego, La Jolla, California, United States of America; 3 Department of Pathology, School of Medicine, University of California San Diego, La Jolla, California, United States of America; 4 Department of Orthopaedic Surgery, Keio University School of Medicine, Tokyo, Japan; 5 Department of Medicine, School of Medicine, University of California at San Diego, La Jolla, California, United States of America; University of California San Francisco, United States of America

## Abstract

Glioblastoma, the most common primary malignant brain tumor, is incurable with current therapies. Genetic and molecular analyses demonstrate that glioblastomas frequently display mutations that activate receptor tyrosine kinase (RTK) and Pi-3 kinase (PI3K) signaling pathways. In *Drosophila melanogaster*, activation of RTK and PI3K pathways in glial progenitor cells creates malignant neoplastic glial tumors that display many features of human glioblastoma. In both human and *Drosophila*, activation of the RTK and PI3K pathways stimulates Akt signaling along with other as-yet-unknown changes that drive oncogenesis. We used this *Drosophila* glioblastoma model to perform a kinome-wide genetic screen for new genes required for RTK- and PI3K-dependent neoplastic transformation. Human orthologs of novel kinases uncovered by these screens were functionally assessed in mammalian glioblastoma models and human tumors. Our results revealed that the atypical kinases RIOK1 and RIOK2 are overexpressed in glioblastoma cells in an Akt-dependent manner. Moreover, we found that overexpressed RIOK2 formed a complex with RIOK1, mTor, and mTor-complex-2 components, and that overexpressed RIOK2 upregulated Akt signaling and promoted tumorigenesis in murine astrocytes. Conversely, reduced expression of RIOK1 or RIOK2 disrupted Akt signaling and caused cell cycle exit, apoptosis, and chemosensitivity in glioblastoma cells by inducing p53 activity through the RpL11-dependent ribosomal stress checkpoint. These results imply that, in glioblastoma cells, constitutive Akt signaling drives RIO kinase overexpression, which creates a feedforward loop that promotes and maintains oncogenic Akt activity through stimulation of mTor signaling. Further study of the RIO kinases as well as other kinases identified in our *Drosophila* screen may reveal new insights into defects underlying glioblastoma and related cancers and may reveal new therapeutic opportunities for these cancers.

## Author Summary

Glioblastomas, the most common primary brain tumor, harbor mutations in receptor tyrosine kinases (RTKs), such as EGFR, and components of the Pi-3 kinase (PI3K) signaling pathway. However, the genes that act downstream of RTK and PI3K signaling to drive glioblastoma remain unclear. To investigate the genetic and molecular basis of this disease, we created a glioblastoma model in the fruit fly *Drosophila melanogaster*. To identify new genes involved in glioblastoma development, we performed a screen for the genes required for tumor cell proliferation using our *Drosophila* glioblastoma model and then functionally assessed the activity of human versions of novel genes identified in this screen. Our results revealed that the RIO kinases become overexpressed in human glioblastomas but not in normal human glial or neuronal cells. We found that overexpression of the RIO kinases promotes and maintains signals that drive tumor cell proliferation and survival in RTK- and PI3K-dependent human glioblastoma, and reduction of RIO kinase expression decreased proliferation and prompted cell death and chemosensitivity in glioblastoma cells. Therefore, disruption of the RIO kinases may provide new therapeutic opportunities to target glioblastoma and other RTK- or PI3K-dependent cancers.

## Introduction

Glioblastoma (GBM), the most common primary malignant brain tumor, infiltrates the brain, grows rapidly, and is refractory to current therapies. Signature genetic lesions in GBM include amplification, mutation, and/or overexpression of receptor tyrosine kinases (RTKs), such as EGFR and PDGFRα, as well as activating mutations in components of the PI-3 kinase (PI3K) pathway (reviewed in [1]). More than 40% of GBMs show EGFR gene amplification, and these amplification events are often accompanied by mutations in EGFR [1]. The most prevalent mutant form of EGFR is ΔEGFR (EGFRvIII, de2-7EGFR, EGFR*), an intragenic truncation mutant that displays constitutive kinase activity [2]. ΔEGFR and other constitutively active mutant forms of EGFR found in GBMs potently drive tumor cell survival, migration, and proliferation [2], [3]. The most frequent mutation in the PI3K pathway in GBM is loss of the PTEN lipid phosphatase, which results in unopposed signaling through PI3K and robust stimulation of Akt, especially in the context of EGFR activation [1]. In mouse models, co-activation of these pathways in glia, glial progenitor cells, and/or neuro-glial stem cells induces GBM [4], [5], [6], [7]. However, the full range of signaling events acting downstream of or in combination with EGFR and PI3K to drive oncogenesis remain to be determined. While several normal effectors of RTK and PI3K signaling, such as Ras, Akt, and mTor, are used by EGFR and PI3K in GBM and are required for gliomagenesis [1], constitutive activation of RTK and PI3K pathways may evoke changes distinct from those induced by normal developmental signaling. Notably, treatments with pharmacologic inhibitors of EGFR or mTor are cytostatic at best in a subset of patients, indicating that other, unidentified factors or compensatory signals affect the survival and growth of tumor cells [8].

To uncover new factors required for EGFR- and PI3K- mediated gliomagenesis, we developed a GBM model in *Drosophila melanogaster* [9]. *Drosophila* offers several advantages for modeling cancers like GBM. Flies have orthologs for 75% of human disease genes [10], including nearly all known gliomagenic genes; signaling pathways are highly conserved; versatile genetic tools are available for cell-type specific gene manipulation [11], ; and *Drosophila* neural cell types are homologous to their mammalian counterparts [13], [14]. While a *Drosophila* model cannot address all aspects of human GBM, our model recapitulates important pathologic features. Specifically, constitutive activation of EGFR-Ras and PI3K signaling in *Drosophila* glial progenitor cells gives rise to proliferative, invasive neoplastic glia that create transplantable malignant tumors [9]. These tumors are induced through activation of a synergistic genetic network composed of downstream pathways commonly mutated and/or activated in human GBMs, such as Akt and mTor signaling [9]. However, activating these known downstream pathways alone or in combination is not sufficient to induce glial neoplasia in *Drosophila*, indicating that additional, as yet unidentified, genetic pathways are involved in transformation. Thus, we undertook genetic screens using our *Drosophila* GBM model to discover new genes underlying EGFR and PI3K mediated neoplastic transformation, and tested whether human orthologs of the genes identified in *Drosophila* represent new human genes involved in GBM.

Our analyses in both *Drosophila* and human systems uncovered that the RIOK1 and RIOK2 kinases drive the survival and proliferation of GBM cells. RIOK1 and RIOK2 are members of the RIO (right open reading frame) family of atypical protein kinases, named for yeast (*S. cerevisiae*) Rio1p and Rio2p, respectively [15]. The RIOK1 and RIOK2 proteins are highly conserved, and are present in all phylogenetic kingdoms, from yeast to mammals among eukaryotes. Kinases in this family are characterized by the presence of the RIO kinase domain, a kinase fold structurally homologous to eukaryotic serine-threonine protein kinase domains, but that lacks classic activation and substrate binding loops (reviewed in [15]). While these kinases undergo autophosphorylation and phosphorylate nonspecific substrates *in vitro*, the actual *in vivo* substrates of RIO kinases are unknown [15]. In both yeast and human cells, RIOK1 and RIOK2, which are not functionally redundant, are required for processing of the 18S rRNA and cytoplasmic maturation of the 40S ribosomal subunit, although neither kinase is an integral component of the ribosome [16], [17], [18], [19]. Recent studies demonstrate that, in yeast, Rio2p also transiently associates with immature ribosomes to block translation initiation, although how RIOK2 is regulated in this context is unclear [20]. To date, several studies have provided suggestive evidence that the RIO kinases could be involved in RTK and PI3K signaling: RIOK2 becomes rapidly phosphorylated in response to EGFR stimulation; Rio2p binds to Tor2p, an ortholog of the mTor kinase, and RIOK1 is required for the proliferation and survival of Ras-dependent cancer cells [21], [22], [23]. However, to date, no specific function has been ascribed to RIOK1 or RIOK2 in the context of EGFR or PI3K signaling.

In this manuscript we demonstrate that RIOK1 and RIOK2 become overexpressed in GBM tumor cells relative to normal brain cells; that RIOK1 and RIOK2 overexpression occurs in response to constitutive Akt signaling; that RIOK2 forms a complex with RIOK1, mTor, and other signaling components to drive activation of Akt signaling and tumorigenesis; and that, in GBM cells, RIOK1 or RIOK2 loss causes a reduction in Akt signaling and provokes p53-dependent apoptosis, cell cycle exit, and chemosensitivity through the RpL11-dependent ribosomal stress checkpoint. Our data demonstrate that the RIO kinases play a key role in Akt-mediated transformation of GBM cells.

## Results

### A kinome-wide screen for modifiers of glial neoplasia

**Figure 1:**
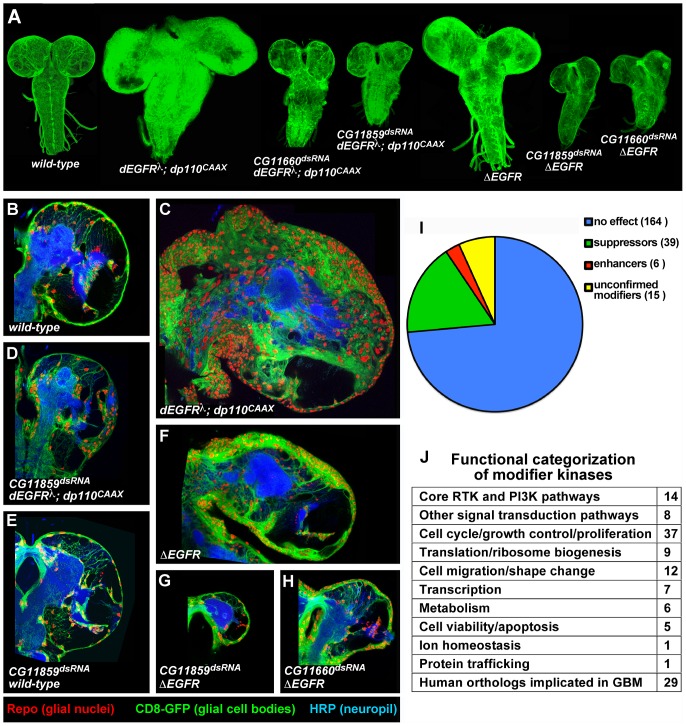
A kinome-wide screen for modifiers of EGFR- and PI3K-dependent glial neoplasia. (A) Optical projections of whole brain-nerve cord complexes from late 3^rd^ instar larvae approximately 130 hrs old, displayed at the same scale. Dorsal view; anterior up. GFP labels glia (green). Each brain is composed of 2 hemispheres and a nerve cord. Knockdown of strong suppressor loci decreased brain size, even relative to wild-type controls, as in *CG11859^dsRNA^;dEGFRλ;dp110^CAAX^* and *CG11660^dsRNA^;dEGFRλ;dp110^CAAX^* animals. Glial-specific overexpression of ΔEGFR drives increased glial cell numbers, brain enlargement, and lethality, and knockdown of strong suppressor loci grossly decreased brain size relative to controls, as in *CG11859^dsRNA^;*Δ*EGFR* and *CG11660^dsRNA^;*Δ*EGFR* animals. (B–H) 3 µm optical projections of brain hemispheres from late 3^rd^ instar larvae approximately 130 hrs old, displayed at the same scale. Frontal sections, midway through brains. Anterior up; midline to left. Glial cell nuclei labeled with Repo (red); glial cell bodies labeled with GFP (green). Brains counter-stained with anti-HRP (blue), which reveals neuropil at high intensity and neuronal cell bodies at low intensity. Dark areas contain unstained neuronal precursor cells. *dEGFRλ;dp110^CAAX^* (C) and *ΔEGFR* (F) brains showed a dramatic increase in glial cell number (red nuclei, green) relative to wild-type (B). Upon suppression, as in *CG11859^dsRNA^;dEGFRλ;dp110^CAAX^* (D), *CG11859^dsRNA^;*Δ*EGFR* (G), and *CG11660^dsRNA^;*Δ*EGFR* (H), there are few excess glia (red nuclei), and remaining glial cells show abnormal development (green). Reduction in both glial (green) and neuronal cell types (low intensity blue) account for reduced brain size upon CG11660 and CG11859 knockdown in the context of *dEGFRλ;dp110^CAAX^* or *ΔEGFR*, which suggests that remaining abnormal glia do not properly support neuronal cell survival. Modifier constructs were also tested for effects in wild-type glia, as in *CG11859^dsRNA^* animals (E). (I) Breakdown of screen results by kinases tested. Unconfirmed modifiers are defined by only one RNAi construct each. (J) Functional classifications of confirmed modifiers. Individual kinases noted in Table S6.

To discover new genes involved in glial pathogenesis, we performed a genetic screen using our *Drosophila* GBM model. Co-overexpression of constitutively active forms of *Drosophila* EGFR (dEGFRλ) and the PI3K catalytic subunit p110α (dp110^CAAX^) stimulates malignant transformation of post-embryonic larval glia, inducing lethal glial neoplasia (Figure 1A–1C) (characterized in detail in [9]). Using this larval *Drosophila* GBM model, we performed an RNAi-based modifier screen for genes that suppress (inhibit) or enhance (worsen) neoplastic phenotypes caused by constitutive EGFR and PI3K signaling. In this scheme, which is an enhancer-suppressor screen, modifier kinases that block/inhibit fly glial neoplasia when their expression is reduced are referred to as ‘suppressors,’ and modifier kinases that exacerbate neoplasia when their expression is reduced are referred to as ‘enhancers.’ This is in keeping with standard *Drosophila* nomenclature, in which genes are classified by their loss-of-function phenotypes. As a side-note, in this context, the term suppressor does not refer to mammalian tumor suppressors.

To enrich for new pathway components, we screened nearly all of the kinases encoded in the *Drosophila* genome (Table S1). Our choice to screen kinases was based on four considerations: (1) kinases regulate a broad array of biological and cellular processes, including those underlying oncogenesis; (2) kinases are highly conserved between *Drosophila* and humans such that every *Drosophila* kinase has a clear human ortholog; (3) as a group, they are well characterized, facilitating functional analysis; and (4) drug discovery efforts are focused on development of specific kinase inhibitors.

We tested 553 conditional RNAi constructs targeting 223 of the 243 kinases in the fly genome (Tables S1, S2) [24], [25]. RNAi constructs were expressed specifically within the glial lineage and were tested for their phenotypic effects on proliferation, migration, morphology, and/or viability of dEGFRλ;dp110^CAAX^ neoplastic glia. The specificity of modifier loci was confirmed by testing multiple RNAi constructs, dominant negative constructs, and/or available mutant alleles to determine if they produced analogous phenotypes in the *dEGFRλ;dp110^CAAX^* model (Tables S2, S3). Control assays were performed for the effects of modifier RNAi constructs when expressed specifically in other cell types, including normal glia, neuroblasts (*Drosophila* neural stem cells), and neurons in order to distinguish those RNAi constructs that caused non-specific toxicity from RNAi constructs that caused specific changes in neoplastic glia (Table S4, Text S1). Constructs that caused early organismal lethality in all cellular contexts tested were excluded from analysis (Tables S2, S4).

To test whether modifiers act in oncogenic signaling downstream of specific EGFR mutations found in human GBM, we created flies that overexpress human ΔEGFR. Glial-specific expression of ΔEGFR caused lethal glial neoplasia phenotypes, alone and in combination with dp110^CAAX^, that were similar to dEGFRλ, and these phenotypes required EGFR kinase activity and core PI3K effectors, such as *dAkt* (Figure 1A–1F, Figure S1, Table S5). We tested modifier RNAi constructs for the ability to alter glial-specific ΔEGFR and *ΔEGFR;dp110^CAAX^* phenotypes (Table S5); the results mirrored their genetic interactions with *dEGFRλ;dp110^CAAX^* (Table S2, S5), indicating that modifiers identified in the screen are common to neoplastic phenotypes conferred by both *Drosophila* and human EGFR.

We identified a total of 45 modifier genes (Figure 1I, Tables S2 and S3). Suppressor RNAi constructs targeting 39 genes reduced neoplasia and induced smaller brain size and lower glial cell numbers relative to *dEGFRλ;dp110^CAAX^* controls, whereas enhancer RNAi constructs targeting 6 genes worsened tumorigenesis and neoplasia and induced increased glial cell numbers, and/or aberrant glial morphologies (Figure 1A–1D, 1I, Tables S2 and S3). Modifiers that suppressed glial neoplasia include genes identified in previous studies, including *dAkt* [9] (Tables S2, S5). A small subset of suppressor constructs caused strong phenotypes in the context of constitutive EGFR-PI3K signaling. Constructs targeting three modifier loci, *Raf*, *Src42A*, and *Taf1*, rescued *dEGFRλ;dp110^CAAX^* animals to adult viability, allowing neoplastic glia to differentiate and function normally despite the presence of dEGFRλ and dp110^CAAX^ (Table S2). Constructs targeting two of the strongest modifiers, *CG11660* and *CG11859* (the *Drosophila* orthologs of the RIOK1 and RIOK2 kinases, respectively), caused severe reduction in brain size and glial cell number when combined with *dEGFRλ;dp110^CAAX^*, *ΔEGFR;dp110^CAAX^*, and/or *ΔEGFR*, as compared to wild-type control animals (Figure 1A–1H, Figure S1). dRIOK2 knockdown gave a stronger effect (Figure 1D, 1G). In contrast, RNAi constructs targeting dRIOK1 and dRIOK2 did not produce a dramatic growth reduction when targeted to normal glia (Figure 1E, Table S4), indicating that dRIOK1 or dRIOK2 knockdown does not simply cause nonspecific cellular toxicity.

Modifier kinases were classified by bioinformatic annotations using Flybase, Gene Ontology, KEGG pathways, the STRING database [26], and comparisons with other *Drosophila* RNAi screens. These classifications (Figure 1J, Figure S2, Table S6) show that kinases with core functions in the RTK and PI3K pathways were highly represented, validating our model and screening methodology [27], [28], [29]. Notably, very few of our modifiers have emerged from *Drosophila* RNAi screens for cell viability (Table S7) [30], indicating that most of the modifiers are not generically required for cell survival. The largest group of modifiers have roles in cell proliferation (Figure 1J, Table S6), and many of these yielded reduced cell lineages upon knockdown in neuroblasts (Table S4) [31], consistent with known requirements for RTK and PI3K signaling in neural progenitor cells [9], [32]. Several of the modifiers involved in cell proliferation, such as *warts* (Table S2), are also components of the hippo pathway, a pathway with a documented role in glial cell proliferation [33]. Broad comparisons with orthologs from species such as yeast (*S. cerevisiae*), revealed modifiers kinases implicated in protein translation, such as dRIOK1 and dRIOK2 [16], [20], or in cell shape change and migration. Finally, comparison to human kinases shows that the majority of our modifier kinases have orthologs previously implicated in GBM (Figure 1J, Figure S2, Table S7), leaving a set of 16 novel modifiers, including dRIOK1 and dRIOK2.

### Overexpression of RIOK kinases in human GBM correlates with Akt activity

Novel modifier kinases identified in our *Drosophila* screens may represent human kinases directly involved in GBM pathogenesis. Kinases that block fly glial neoplasia when their expression is reduced are of interest because their human orthologs may be promising new targets for therapeutic inhibition. There are 27 human orthologs for the 16 novel *Drosophila* modifier kinases (Table S8). To determine if any of these human kinases are expressed or mutated in GBM, we analyzed tumor genomic databases, proteomic atlases, and GBM cell lines, which provided suggestive evidence that 12 modifier orthologs are subject to genetic alteration and/or elevated gene or protein expression in GBMs (see Text S1, Tables S9 and S10, Figures S3 and S4). Among these, RIOK1 and RIOK2 showed increased protein expression consistent with involvement in GBM. Given that loss of the dRIOKs strongly and specifically blocks growth and survival of EGFR and PI3K mutant glia, and that recent publications suggest that the RIO kinases may contribute to EGFR and/or mTor signaling [21], [22], the functional roles of RIOK1 and RIOK2 in GBM were of particular interest.

**Figure 2:**
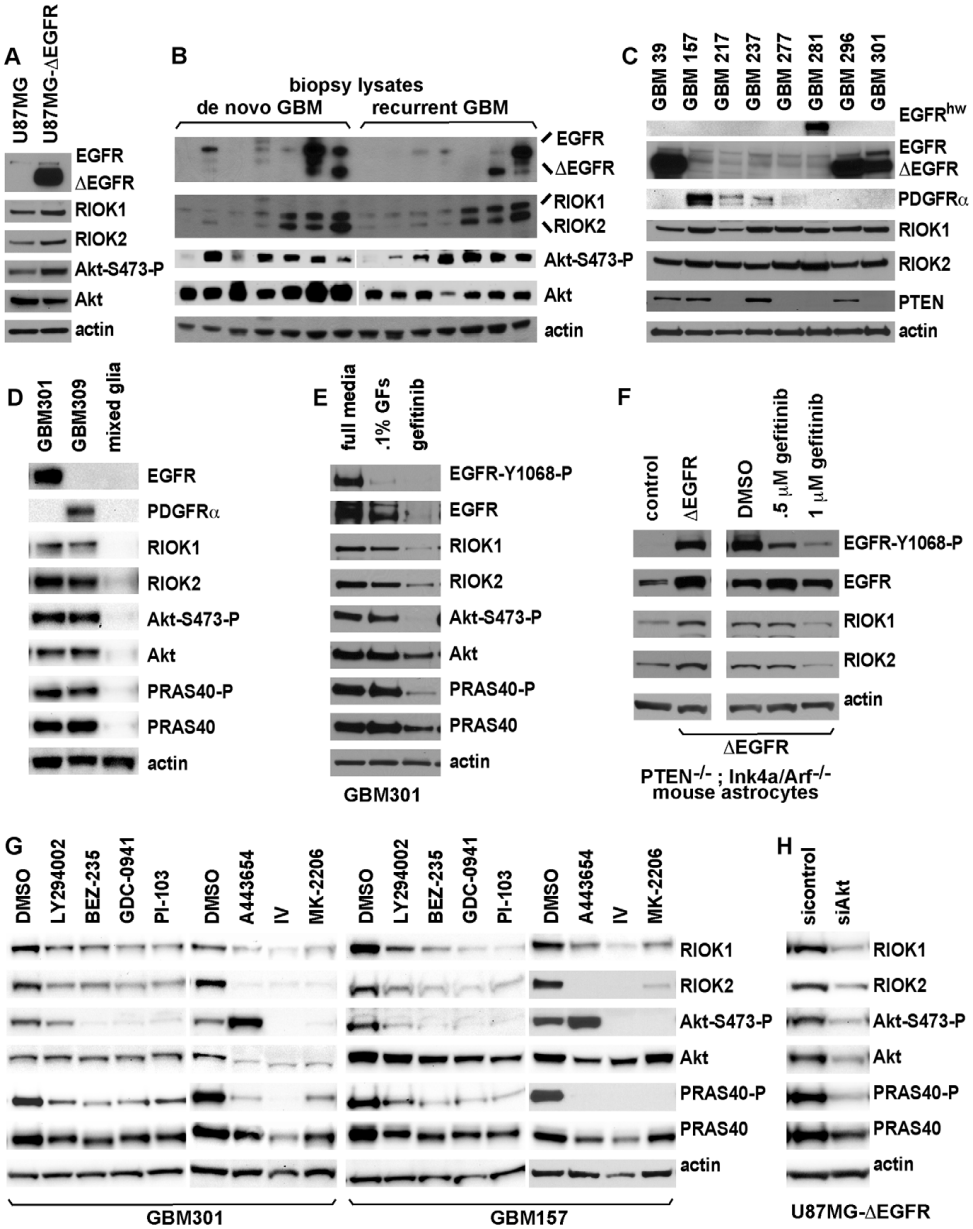
Expression of RIO kinases is associated with EGFR and Akt activity in GBM cells. (A) U87MG and U87MG-ΔEGFR cells cultured with .1% serum for 36 hrs to enrich ΔEGFR signaling. ΔEGFR runs below full-length EGFR. (B) Biopsies of new (de novo) and recurrent GBMs. RIOK1 (∼75 kDa) and RIOK2 (∼63 kDa) antibodies used serially on the same blot. Serine-473 phosphorylation is a proxy for Akt activation. (C) Primary neurosphere GBM cultures. GBM39 is isolated from a ΔEGFR-positive serial xenograft [41]. Others are low-passage cultures established from fresh tumors; expression of ΔEGFR, EGFR, or PDFGRα derives from parent tumors [35]. EGFR^hw^ is a high molecular weight (>200 kDa) mutant version detected in GBM 281. (D) RIOK expression and Akt signaling in neurospheres compared with a fresh culture of mixed human glia and astrocytes (established from normal adult cortex) grown under the same conditions. (E) GBM301 treated for 24 hrs with growth factor withdrawl (.1% GFs, .1% of the normal growth factor dosage) or 5 µM gefitinib. GBM301 cells are ΔEGFR-positive, EGFR-amplified, and PTEN-negative (see C). (F) Extracts from *PTEN^−/−^; Ink4a/arf^−/−^* astrocytes transduced with empty vector (left) or ΔEGFR (right), or grown with .5% serum and treated with gefitinib for 24 hrs (far right). EGFR inhibition evidenced by reduced Tyrosine-1068 phosphorylation. (G) Neurosphere cultures treated for 24 hours with DMSO or indicated inhibitors. P110 inhibitors: 50 µM LY294002, 1 µM BEZ-235, 2 µM GDC-0941 [71], 2 µM PI-103 [71]. Akt inhibitors: 1 µM A443654, 10 µM Akt inhibitor IV [40], and 8 µM MK-2206 [72]. Inhibition of PI3K-Akt signaling evidenced by reduced phosphorylation of PRAS40, a direct Akt substrate, and reduced Akt and/or PRAS40 protein levels. With A443654, increased Akt-Ser473 phosphorylation occurs despite Akt inhibition [40]; decreased Akt-Ser473 phosphorylation occurs with Akt inhibitor IV and MK-2206 [72]. (H) U87MG-ΔEGFR cells treated with pan-Akt siRNAs compared to cells treated with nontargeting control siRNAs, harvested 72 hours post-transfection.

A range of GBM cells and cell lines were examined to determine how RIOK1 and RIOK2 expression correlated with tumor cell genotype and phenotype. Our analyses showed that RIOK1 and RIOK2 were expressed in PTEN-null U87MG GBM cells and were upregulated in U87MG cells engineered to express ΔEGFR at levels detected in tumors [34] (Figure 2A). RIOK1 and RIOK2 were also upregulated in GBM tumors with EGFR overexpression/mutation as well as activated Akt (Figure 2B), although these correlations were not clear in all specimens. Primary neurosphere cultures, which are composed of neural stem cell-like human GBM cells propagated in EGF-supplemented media [35], [36], [37], can maintain mutations/gene expression found in their parent tumors [37]. Neurosphere cultures showed strong RIOK1 and RIOK2 expression (Figure 2C); these included neurosphere lines with ΔEGFR, as well as neurosphere lines displaying PDGFRα overexpression, PTEN loss, and/or other mutant forms of EGFR (Figure 2C). In a panel of standard GBM cell lines, RIOK1 and RIOK2 showed strong expression in cell lines known to harbor PTEN and/or EGFR mutations, and RIOK1 and RIOK2 expression was comparatively lower in a GBM cell line with intact PTEN (Figure S5) [38], [39]. In contrast, RIOK1 and RIOK2 were nearly undetectable in mixed glial cultures freshly derived from adult human cortex (Figure 2D). Thus, RIOK1 and RIOK2 overexpression appeared to be correlated with RTK mutation/overexpression and/or PTEN loss in GBM tumor cells.

To determine if elevated RIOK1 and/or RIOK2 expression in GBM cells depends on EGFR and/or PI3K signaling, neurosphere lines and U87MG-ΔEGFR cells were treated with relevant inhibitors (Figure 2E and 2G, Figure S6). RIOK1 and RIOK2 levels decreased upon either growth factor withdrawal or gefitinib treatment of primary neurosphere cultures (Figure 2E), indicating that their up-regulation can be EGFR-dependent. Consistent with this, *Pten^−/−^; Ink4a/arf^−/−^* mouse astrocytes transformed by ΔEGFR showed increased RIOK1 and RIOK2 levels, which were reduced by gefitinib treatment (Figure 2F). RIOK1 and RIOK2 protein levels also decreased in neurosphere cells and U87MG-ΔEGFR cells treated with inhibitors of the p110 PI3K catalytic subunit, such as BEZ-235, and inhibitors of Akt, such as A443654 (Figure 2G) [40]. siRNA-mediated Akt knockdown or restoration of PTEN function in U87MG-ΔEGFR cells also reduced RIOK protein levels (Figure 2H, Figure S7). Treatments with p110 and Akt inhibitors also demonstrated that p110 and Akt signaling is required for RIOK1 and RIOK2 expression in PDGFRα-overexpressing neurospheres (Figure 2G). Taken together, these data indicate that RIOK1 and RIOK2 overexpression in GBM cells is driven by Akt activity downstream of RTK mutation/overexpression and/or PTEN loss.

However, the role of factors that act downstream of Akt was less clear (mTor inhibitors did not always reduce RIOK levels, see Figure S6), suggesting that RIOK1 or RIOK2 levels may be directly regulated by Akt. Given that mRNA expression levels of RIOK1 and RIOK2 did not show significant upregulation in tumor samples with PTEN and/or EGFR alterations (Table S9), and given that RIOK1 and/or RIOK2 levels decline after short-term treatments with Akt inhibitors (Figure S7), we hypothesized that Akt signaling may regulate RIOK2 and/or RIOK1 levels by modulating protein stability post-translationally. Consistent with this, addition of a proteosome inhibitor, MG132, prevented the reduction in RIOK1 and/or RIOK2 protein levels observed upon A443654 treatment or PTEN add-back (Figure S7). RIOK2 has several mapped serine phosphorylation sites (www.phosphosite.org), including putative Akt target sites (Figure S8). However, mutation of this single site did not abolish detection of RIOK2 by a phospho-Akt-substrate antibody (Figure S9). Thus, Akt-mediated regulation of RIO kinase levels does not hinge on phosphorylation at a single residue in RIOK2, and likely involves a more complex mechanism that requires more investigation.

**Figure 3:**
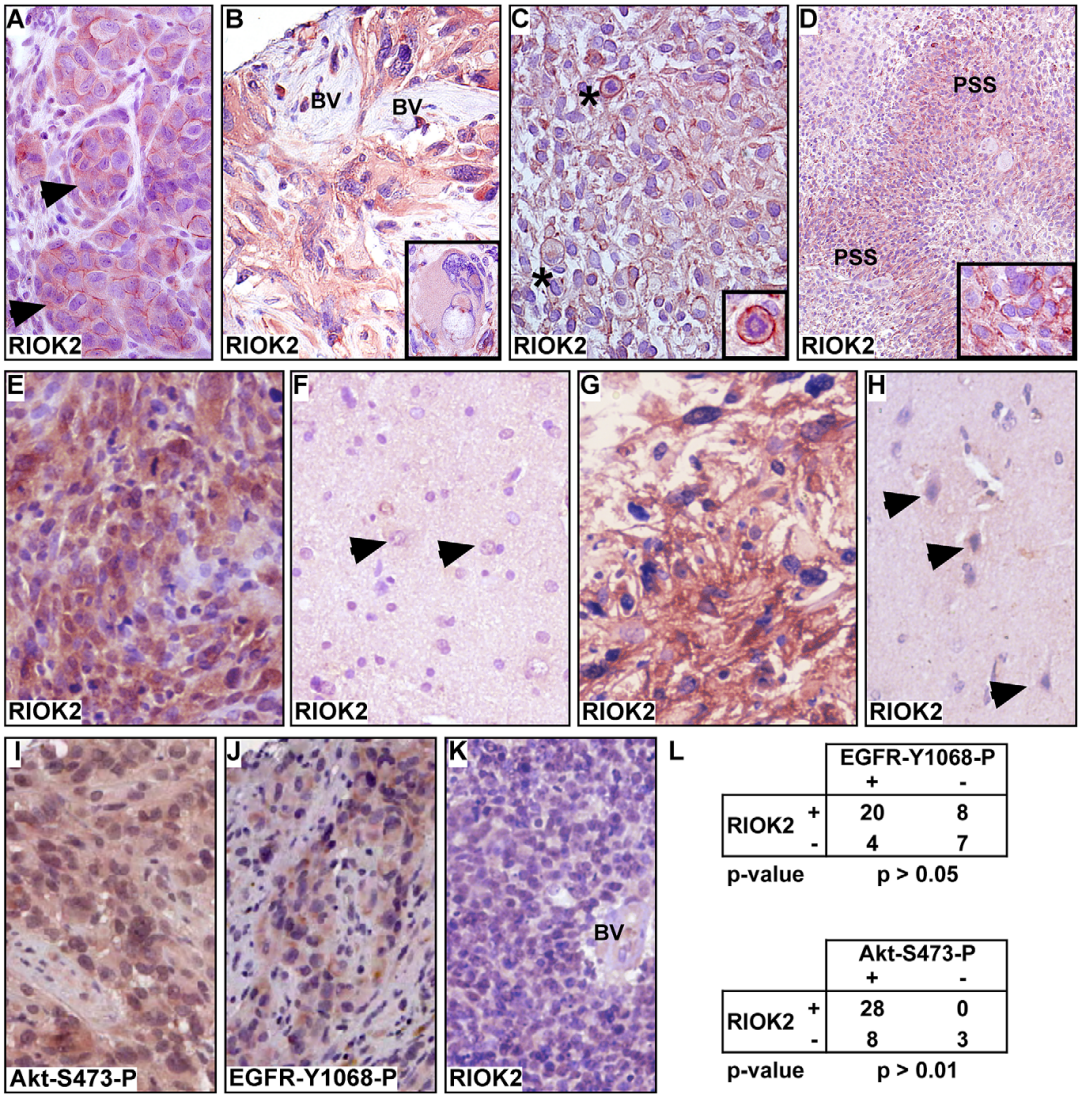
RIOK2 overexpression in GBM tumors is associated with Akt signaling. (A–E) Immunohistochemistry for RIOK2 (reddish brown) showing cytoplasmic and submembraneous enrichment for RIOK2 in tumor cells. Hematoxilin counterstain, (A) GBM39 tissue, from a subcutanteous xenograft, showing RIOK2 staining in tumor cells (arrows), which formed lobules delineated by RIOK2-negative host stromal cells. (B) ΔEGFR-positive human GBM with RIOK2-positive giant cell component (inset shows a conspicuous giant cell), and RIOK2-negative tumor stroma composed of abnormal blood vessels (“BV”). (C) ΔEGFR-positive human GBM, abnormal mitotic cells with high RIOK2 staining denoted with asterisks and shown in inset close-up. (D) ΔEGFR-positive human GBM, lower magnification to highlight enriched RIOK2 in pseudopallisades (“PSS”), inset shows enriched RIOK2 staining present in dense cellular regions of pseudopallisades. (E) RIOK2 expression in an EGFR-overexpressing human GBM with (F) matched normal control tissue from the same surgical specimen, arrows denote normal astrocytes (recognized by their open nuclei). (G) RIOK2 expression in an EGFR-negative/Akt-S473-P-positive GBM shown alongside (H) another example of normal control brain tissue. Arrows denote normal neuronal cells (recognized by their basophilic cell bodies) with low/undetectable RIOK2 expression. (I) and (J) examples of Akt-S473-P and EGFR-Y1068-P immunoreactivity in RIOK2-positive GBM tumor specimens. (K) a RIOK2- negative GBM with a negative abnormal blood vessel (“BV”). (L) Statistical analysis of RIOK2-positive and negative tumor specimens showing a significant correlation between RIOK2 expression and phosphorylation of EGFR at Tyrosine-1068 and phosphorylation of Akt at Serine-473. More stains from tumors shown in Figures S10 and S11.

To confirm that the RIOKs are expressed in GBM, we performed immunohistochemistry (IHC) for RIOK2 on a group of typed tumor specimens (RIOK1 antibodies were unsuitable for IHC). Xenograft specimens of GBM39, which is ΔEGFR-positive [41], showed RIOK2 expression in tumor cells, with cells displaying diffuse cytoplasmic and sub-surface RIOK2 localization (Figure 3A). In contrast, murine stromal cells had little or no RIOK2 immunoreactivity, although the antibody can detect mouse RIOK2. ΔEGFR-positive and EGFR-overexpressing specimens displayed strong, but sometimes heterogeneous, cytoplasmic RIOK2 immunoreactivity, ranging from the giant cell to the small cell populations (Figure 3B–3E, Figures S10 and S11), with the strongest expression in mitotic cells and densely cellular pseudopallisades (Figure 3C and 3D, Figure S10). Heterogeneity in RIOK2 expression may possibly reflect heterogeneity of RTK expression in tumors [42], [43], or may reflect upregulation of RIOK2 in actively cycling cells given the increased immunoreactivity observed in mitotic cells. Cytoplasmic localization of RIOK2 in GBM is consistent with observations of RIO kinase localization in yeast and human cells [16], [17], [18]. In contrast, RIOK2 did not show appreciable immunoreactivity in neural cells in matched normal control brain (n = 14) (Figure 3F, 3H), and did not show immunoreactivity in tumor stroma (Figure 3B), demonstrating that RIOK2 upregulation is tumor-specific. Akt signaling in tumors was assessed by staining for Akt phosphorylated at Serine-473 (Akt-S473-P, example shown in Figure 3I), and EGFR status of tumors was primarily assessed with staining for EGFR phosphorylated on Tyrosine-1068 (EGFR-Y1068-P, example shown in Figure 3J), which indicates EGFR activation. Statistical analysis demonstrated that RIOK2 expression was significantly correlated with EGFR status in tumor specimens (Figure 3L), although some EGFR-negative tumors also showed RIOK2 immunoreactivity (Figure 3G, 3L), while some EGFR-negative tumors did not (Figure 3K). The correlation of RIOK2 expression with EGFR activity is likely secondary to Akt-mediated regulation of RIOK2: RIOK2-expressing specimens positive EGFR-Y1068-P always showed staining for Akt-S473-P (n = 20). Indeed, all specimens that showed RIOK2 immunoreactivity, whether EGFR-positive or EGFR-negative, showed staining for Akt-S473-P (Figure 3L).

### RIOK2 overexpression in astrocytes induces invasive glial tumors and TORC2-Akt activation

**Figure 4:**
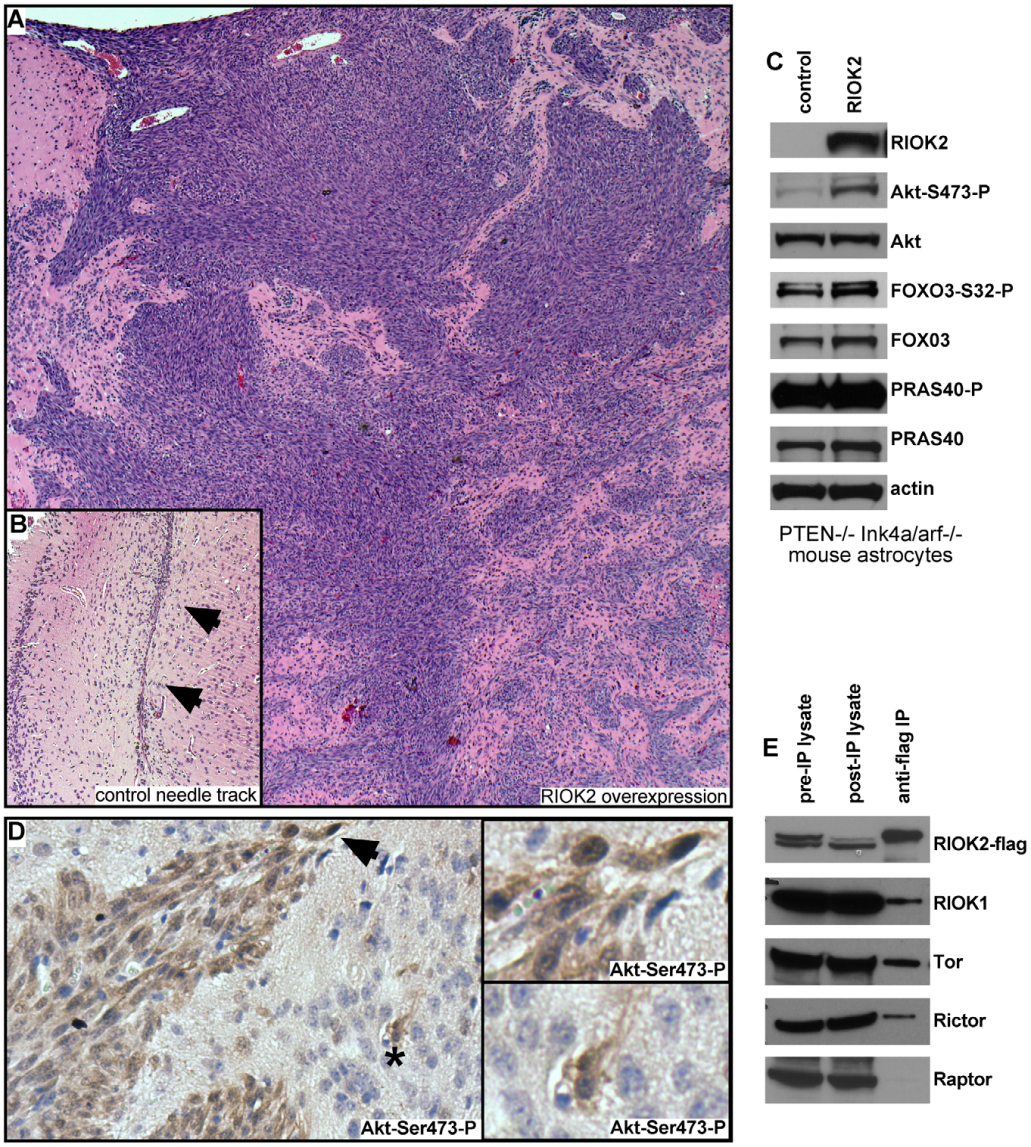
Overexpression of RIOK2 in murine astrocytes promotes tumorigenesis and TORC2-Akt signaling. (A) H&E stain showing high-grade glioma derived from *RIOK2^overexpression^; Pten^−/−^; Ink4a/arf^−/−^* astrocytes grafted into the mouse brain. Tumor cells (purple) generate masses composed of spindle-shaped cells as well as infiltrative neoplastic cells that show invasion into the parenchyma and along blood vessels, animals sacrificed ∼19 days following injection. (B) representative needle tract (arrows) in a mouse brain grafted with control *Pten^−/−^; Ink4a/arf^−/−^* astrocytes engineered with empty vector; note the slight concentration of astrocytic cells along the needle tract but no tumor mass or infiltrates. (C) Western blots of *RIOK2^overexpression^; Pten^−/−^; Ink4a/arf^−/−^* astrocytes compared to *Pten^−/−^; Ink4a/arf^−/−^* astrocytes with empty vector, grown *in vitro*. (D) Immunoreactivity for Akt phosphorylated at Serine-473 (reddish brown) in a tumor derived from *RIOK2^overexpression^; Pten^−/−^; Ink4a/arf^−/−^* astrocytes, tumor margin shown, with many surrounding normal cells (purple nuclei, faint staining). Arrow indicates strong staining in invasive cells at tumor margin, asterisk indicates more distant individual invasive cells; both shown in close-up (right). (E) Epitope tagged RIOK2 (RIOK2-flag, runs slightly larger than endogenous untagged RIOK2) was overexpressed in 293T cells and immunoprecipitated along with associated proteins. Blots were probed for indicated proteins, whole lysates from both before (pre-IP) and after (post-IP) are included as a control for protein expression.

To determine if elevated levels of RIOK2 drives oncogenic changes in mammalian cells, we tested the effects of RIOK2 overexpression in astrocytes. *Pten^−/−^; Ink4a/arf^−/−^* murine astrocytes, which are immortalized by tumor suppressor mutations common in GBM, express little endogenous RIOK2 and are not gliomagenic in intracranial grafts assays [44]. In two experiments, mice intracranially grafted with *Pten^−/−^; Ink4a/arf^−/−^* astrocytes overexpressing RIOK2 showed symptoms of hydrocephaly and neurological deficits 3 weeks following implantation, unlike mice grafted with *Pten^−/−^; Ink4a/arf^−/−^* control astrocytes. Histological analysis showed that control *Pten^−/−^; Ink4a/arf^−/−^* astrocytes yielded no intracranial tumors in 12 total animals tested, whereas *RIOK2^overexpresion^; Pten^−/−^; Ink4a/arf^−/−^* astrocytes formed invasive high-grade glial tumors composed of invasive spindle-shaped cells in 7 out of 10 total animals tested (p<.001 by chi-squared test) (Figure 4A, 4B).

In GBM, ΔEGFR drives strong Akt activation in the context of PTEN loss, and can drive gliomagenic transformation of *Pten^−/−^; Ink4a/arf^−/−^* astrocytes [6], [45]. Thus, we wondered whether RIOK2 overexpression also drives astrocyte transformation by activating Akt. Consistent with this, *RIOK2^overexpression^; Pten^−/−^; Ink4a/arf^−/−^* astrocytes displayed increased phosphorylation of Akt at Serine-473 (Figure 4C), and tumor tissue from *RIOK2^overexpression^; Pten^−/−^; Ink4a/arf^−/−^* cells showed specific staining for Akt-Ser473-P (Figure 4D). Phosphorylation of Serine-473 is required for Akt activity towards select substrates such as FOXO3 [46], a direct Akt substrate that governs GBM cell tumorigenicity [47]. FOXO3 also displayed increased phosphorylation *RIOK2^overexpression^; Pten^−/−^; Ink4a/arf^−/−^* astrocytes (Figure 4C). However, we did not observe increased phosphorylation of all Akt targets, including PRAS40, with RIOK2 overexpression, suggesting that the effect of RIOK2 on phosphorylation of Akt targets was selective to Serine-473 dependent substrates [46].

Akt is phosphorylated on Serine-473 by mTor-complex-2 (TORC2), a multi-protein complex composed of the mTor kinase and several other signaling components, including Rictor [46], a protein that becomes elevated in glioblastomas that also drives gliomagenesis when overexpressed in astrocytic cells [48], [49]. In yeast proteomic analyses, Rio2p has been shown to bind to Rio1p and Tor2, the yeast mTor ortholog that forms the equivalent of TORC2 [22]. From human cells overexpressing RIOK2, RIOK2 co-immunoprecipitated with RIOK1, mTor, and Rictor, a protein which is definitive of the TORC2 complex (Figure 4E) [46]. mTor also associates with another complex, mTor-complex-1 (TORC1), which phosphorylates other mTor substrates, such as EIF-4E, and is composed of signaling components including the Raptor protein [46]. Raptor did not co-immunoprecipitate in the RIOK2-RIOK1-mTor-Rictor complex (Figure 4E), suggesting that RIOK2 specifically associates with TORC2. Taken together, these data suggest that RIOK2 directly binds to TORC2 to stimulate phosphorylation of Akt at Serine-473 and activation of Akt towards select substrates, such as FOXO3, and that this process may directly involve RIOK1 recruitment.

### Requirement for RIOK kinases for proliferation and survival in GBM cells

**Figure 5:**
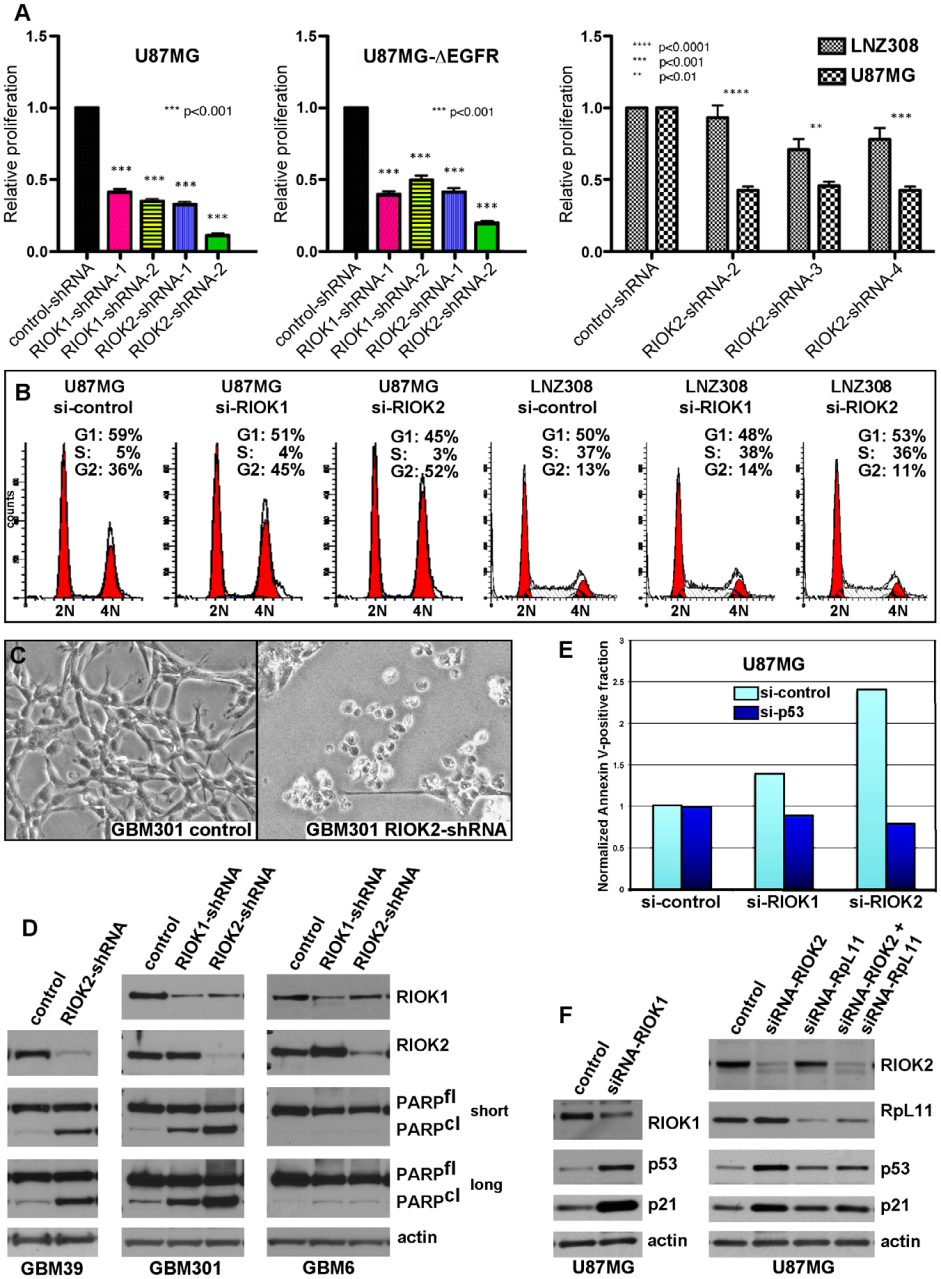
RIOKs drive proliferation and survival of GBM cells in a p53-dependent manner.

**Figure 6:**
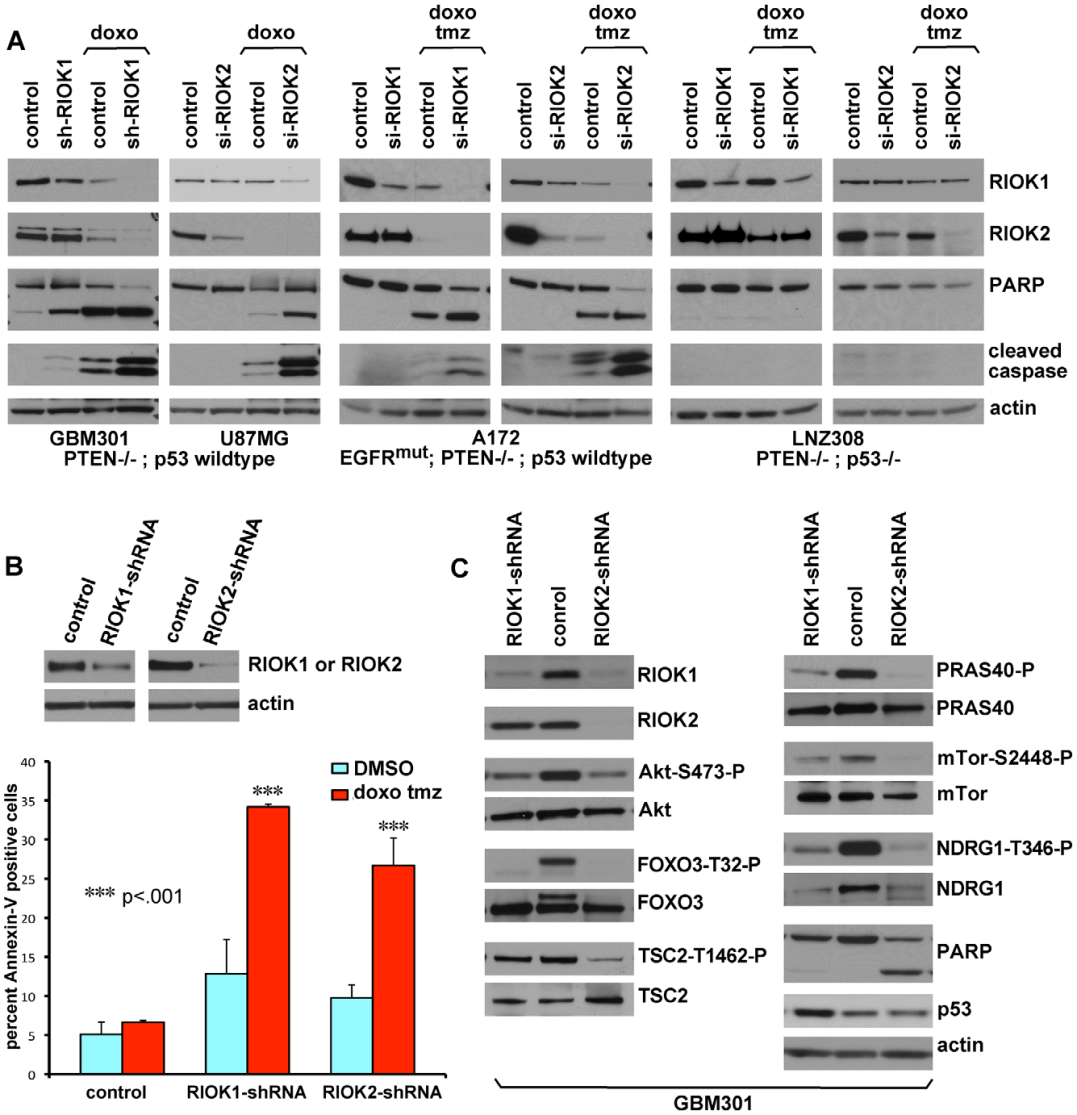
Loss of RIOK1 or RIOK2 function chemosensitizes GBM cells and reduces TORC2-Akt signaling. (A) Knockdown of RIOK1 or RIOK2 sensitizes GBM cells to apoptosis in response to treatment with doxorubicin (doxo) and temozolomide (tmz), as evidenced by blots for active caspase-3 and PARP cleavage (A). All samples blotted for RIOK1 and RIOK2 to confirm changes in RIOK1 levels with RIOK2 knockdown, evident in p53-wild-type GBM cell lines. The RIOKs also decline with doxorubicin treatment. GBM301 cells were treated for 24 hrs with 1 µg/mL doxorubicin beginning 96 hrs post infection with viral vectors. U87MG, A172, and LNZ308 cells were treated for 24 hrs with 1 µg/mL doxorubicin and 100 µM temozolomide beginning 72 hrs post transfection with siRNAs. (B) FACS-based quantification of chemosensitivity. 96 hours post shRNA infection, U87MG samples were split in half and treated for 12 hours with either DMSO (light blue) or 1 µg/mL doxorubicin and 100 µM temozolomide (red). Live cells were collected and stained for 7AAD and Annexin-V. Data is represented as the percentage of Annexin V-positive 7AAD-negative cells in each sample, averaged over 2 experiments. P-values refer to student's two-tailed t-test used to compare doxorubicin and temozolomide-treated control to RIOK-shRNA cells. Validation of knockdown shown. FACS plots and raw data shown in Figure S15. (C) GBM301 cells treated with 25 µM ZVAD for 48 hrs beginning 3 days post-infection with viral vectors. Reduced phosphorylation of Akt on the TORC2 target site, Serine-473, is visible relative to total Akt protein. Reduced phosphorylation of several Akt targets, such as the FOXO3 transcription factor, is clear when phospho-epitope signal is compared to total protein controls. PARP cleavage is a read-out for apoptosis; PARP cleavage fragment in RIOK2 knockdown cells indicates residual caspase activity, due to the strong effect of RIOK2 loss. p53 upregulation was evident in GBM301 cells in the absence of residual caspase activity.

Given that knockdown of their *Drosophila* cognates yields growth reduction of neoplastic glia, we tested RNAi constructs targeting human orthologs of novel suppressor kinases for their requirement in GBM cell survival and proliferation (Text S1, Figure S12). Among these, RIOK1 or RIOK2 knockdown yielded strong effects, inhibiting U87MG-ΔEGFR and U87MG proliferation (Figure 5A, 5B). ΔEGFR-positive neurosphere cultures, such as GBM301 and GBM39, also showed a pronounced apoptotic response to RIOK2 or RIOK1 knockdown, with RIOK2 loss yielding stronger effects (Figure 5C, 5D). In U87MG cells, which are dependent on Akt signaling for growth [50], RIOK1 or RIOK2 RNAi provoked G2 cell cycle arrest and reduced proliferation (Figure 5A–5B). The phenotypes caused by RIOK1 and RIOK2 knockdown were observed in other GBM cell lines that are PTEN and/or EGFR mutant, such as A172 (Figure 6, Figure S14, data not shown). Of note, RIOK2 knockdown typically triggered a reduction in RIOK1 expression, regardless of the RIOK2 RNAi constructs used, suggesting that RIOK2 regulates RIOK1 protein levels (Figure 5D, Figure S13, see also Figure 6).

Given that the RIO kinases have been found to stimulate ribosome maturation, we initially suspected that functional reduction of RIOK1 or RIOK2 may cause generic cellular toxicity. However, in testing multiple GBM cell lines that showed strong RIOK expression, we found that a subset of GBM cells were far less affected by knockdown of RIOK1 and RIOK2. GBM6, a ΔEGFR-positive neurosphere line, did not undergo apoptosis upon RIOK1 or RIOK2 knockdown (Figure 5D). Moreover, LNZ308 cells, which are PTEN mutant [38], did not show cell cycle defects or strong reduction of RIOK1 expression with RIOK2 knockdown (Figure 5B). Both GBM6 and LNZ308 are also mutant or null for p53, whereas GBM cells that show cell cycle defects and apoptosis upon RIOK loss, such as U87MG, are wild-type for p53 [38], [41]. We observed a similar lack of apoptosis upon RIOK1/RIOK2 loss in other p53 mutant/null GBM cells, such as U373 (data not shown). This implies that the survival and proliferation defects induced by RIOK1 and RIOK2 loss rely on p53. Consistent with this, concomitant knockdown of p53 with RIOK1 or RIOK2 in U87MG cells blocks the apoptosis observed upon RIOK2 or RIOK1 knockdown alone (Figure 5E).

In human cells, RIOK1 and RIOK2 transiently associate with immature cytoplasmic 40S ribosomal subunits to promote their maturation and stimulate rRNA processing, like their yeast counterparts [18], [19], [22], [51]. Defects in ribosome biogenesis and rRNA processing can activate a p53-dependent ribosomal-stress checkpoint to suppress growth and induce cell cycle arrest and apoptosis, a process that relies on p53 upregulation and transcriptional activation mediated by release of the RpL11 ribosomal protein (reviewed in [52], [53]). In U87MG cells and other GBM cell lines, RIOK1 or RIOK2 knockdown induced up-regulation of p53 and the p21 cdk inhibitor, a p53 transcriptional target (Figure 5F, Figure S13, S14), and coincident knockdown of RpL11 and RIOK1 or RIOK2 blocked induction of p53 and p21 (Figure 5F and Figure S14). Therefore, RIOK1 or RIOK2 loss leads to p53 activation, which requires the p53-RpL11-dependent ribosomal stress checkpoint.

### Loss of RIOK function chemosensitizes GBM cells in a p53-dependent manner

p53 is often downregulated in GBM tumors and tumor cells with activated Akt, PTEN loss, and/or EGFR mutation/overexpression [54]. Yet, the majority of tumors with PTEN loss or EGFR mutation/amplification have intact p53 loci (EGFR: 85–92%, PTEN: 92–95%) [55], [56]. Given that RIOK loss upregulates p53 levels, we tested whether knockdown of RIO kinases could potentiate the response of GBM cells to treatments with DNA-damaging agents such as doxorubicin, which cooperates with p53 to provoke apoptosis, [57], [58] and temozolomide, which is a DNA alkylator used to treat GBM. In GBM cells wild-type for p53, such as GBM301 and U87MG, knockdown of RIOK1 or RIOK2 potentiated apoptotic responses to doxorubicin and/or temozolomide (Figure 6A–6B, Figure S15). In contrast, cells mutant for p53, such as LNZ308 cells, did not show apoptosis upon RIOK1 or RIOK2 knockdown and doxorubicin-temozolomide treatments (Figure 6A). Therefore, inhibition of the RIO kinases chemosensitizes EGFR- and/or PTEN mutant GBM cells.

Our results suggest that elevated p53 activity can potentiate elimination of EGFR and/or PTEN mutant GBM cells. One way to increase p53 levels and activity is with nutlin-3, a small molecule which is know to cause cell cycle arrest and sensitivity to DNA-damaging agents in U87MG cells [59]. However, nutlin-3 did not provoke the same cell cycle defects observed with RIO kinase knockdown, despite inducing high levels of p53 and p21 (Figure S16). Thus, other changes induced by RIO kinase loss must contribute to cell cycle arrest and apoptosis.

### Loss of RIOK function antagonizes Akt signaling

**Figure 7:**
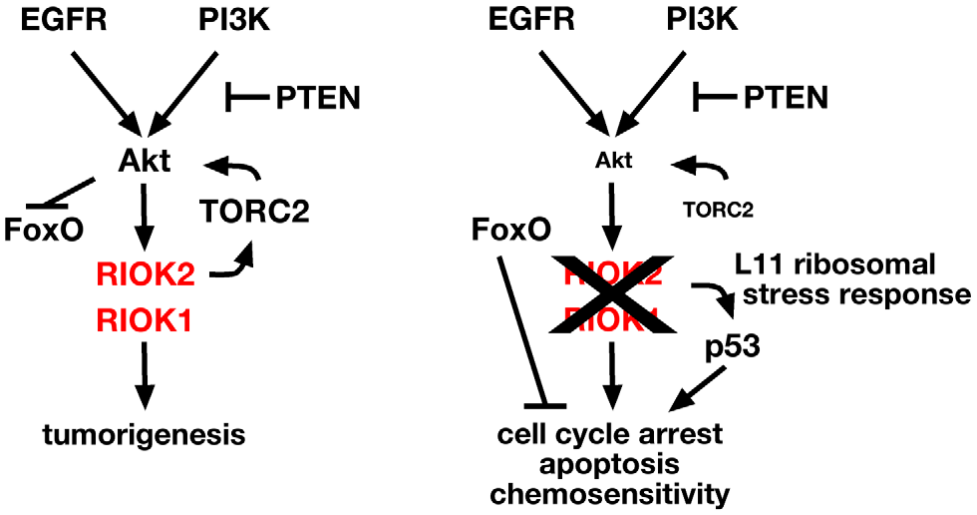
RIOK1 and RIOK2 are required for EGFR- and PI3K-mediated tumorigenesis. Pathway diagram placing RIOK1 and RIOK2 in relation to Akt downstream of EGFR and PI3K signaling in GBM. RIOK2 mediates signaling both upstream and downstream of Akt via stimulation of TORC2 (left). Loss of RIOK1 or RIOK2 reduces Akt signaling downstream of oncogenic EGFR and PI3K signaling, and induces the p53-dependent ribosomal stress checkpoint via RpL11 (right).

We tested for signaling alterations that occur upon RIOK1 or RIOK2 knockdown that would explain reduced proliferation and survival of GBM cells. The caspase inhibitor ZVAD was used to dampen apoptosis and thus preserve signaling defects. Compared to controls, RIOK1 or RIOK2 knockdown led to reduced phosphorylation of Akt at Serine-473 and reduced phosphorylation of Akt target proteins such as FOXO3 (Figure 6C, Figure S17). This occurred in both p53 wild-type and p53 mutant GBM cells (Figure 6C, Figure S17). Serine-473 is phosphorylated by Tor complex 2, (TORC2) [46], and in yeast and human cancer cells, TORC2 phosphorylation of Akt is stimulated by mature ribosomes, which can bind to both TORC2 and Akt to mediate their interaction, and TORC2 activity is blocked by defects in ribosome biogenesis [60]. Given that RIOK1 and RIOK2 loss causes defects in 40S ribosome maturation [17], [18], [19], and that we discovered that RIOK1 and RIOK2 bind to TORC2 components, we hypothesized that RIOK1 and RIOK2 knockdown interferes with TORC2 activity. Consistent with this, other readouts of TORC2 activity, such as phosphorylation and levels of NDRG1 [60], were reduced (Figure 6C, Figure S17), demonstrating that TORC2 activity is downregulated by RIOK1 and RIOK2 loss. This is consistent with recent findings demonstrating a requirement for TORC2 signaling in Drosophila glial neoplasia as well as human GBM cells [9], [48], [49]. However, the effects of RIOK loss on Akt signaling were not limited to the TORC2-dependent substrates. Phosphorylation of other Akt substrates, such as PRAS40 and TSC2, can also be reduced upon RIO kinase knockdown (Figure 6C). Thus, RIOK1 and RIOK2 are necessary for Akt signaling in GBM cells. Over-all, our results strongly suggest that functional reduction of RIOK1 and RIOK2 results in loss of Akt activity and p53 activation to drive cell cycle arrest, chemosensitivity, and apoptosis in Akt-dependent GBM cells with intact p53 (Figure 7).

## Discussion

From a *Drosophila* genetic screen, we identified genes encoding 16 novel kinases that affect EGFR- and PI3K- dependent neoplastic glial transformation. We found that a subset of human orthologs for these novel kinases, including RIOK1 and RIOK2, are subject to alterations in GBM. RIOK1 and RIOK2, two related and highly conserved atypical kinases, become upregulated in an Akt-dependent manner in GBM cells. Our results show that RIOK2 forms a complex with RIOK1 and TORC2 signaling components, drives activation of TORC2-dependent Akt signaling, and stimulates glial tumorigenesis. Furthermore, we found that, in GBM cells, RIOK1 or RIOK2 loss causes a reduction in Akt signaling towards TORC2-depdendent targets and provokes p53-dependent apoptosis, cell cycle exit, and chemosensitivity. Thus, our loss-of-function and gain-of-function data imply that RIOK2 creates a feedforward loop that promotes and maintains Akt activity, and disruption of this loop is sufficient to trigger chemosensitivity and apoptosis in Akt-dependent GBM cells with intact p53 (Figure 7). Our results may have broad relevance to other cancers since RIOK2 is strongly expressed in a range of other more common tumor types associated with high Akt activity, such as breast and prostate cancers (Figure S18). Further study of the RIO kinases as well as other kinases identified in our *Drosophila* screen may reveal new insights into the signaling defects underlying GBM and related cancers.

RIOK1 and RIOK2 upregulation was associated with Akt activity in both GBM tumor specimens and cultured cells, and our results show that Akt signaling regulates RIO kinase protein stability, although the exact mechanism by which Akt regulates RIO kinase levels remains undetermined. RIOK2 has several putative and mapped phosphorylation sites, including at least one putative Akt phosphorylation site (www.phosphosite.org, Figure S8). Other studies show that RIOK2 phosphorylation can be stimulated by EGFR, and can be carried out by Polo-like kinase 1 [21], [61], and perhaps these events contribute to Akt-mediated regulation of RIO kinase levels. Of note, though standard GBM cells lacking PTEN showed high levels of RIO kinase expression, non-transformed astrocytes lacking PTEN did not show high levels of endogenous RIO kinase protein expression relative to astrocytes with intact PTEN. Therefore, other factors present in GBM cells must also contribute to elevated RIO kinase levels.

To date, published studies show that the RIO kinases act as ribosome assembly factors that transiently associate with the 40S subunit to promote ribosome maturation and translation initiation [17], [18], [20]. Given that mature ribosomes are required for TORC2 activation and Akt phosphorylation at Serine-473 [60], disruption of Akt signaling upon RIOK knockdown may be a result of defective ribosome biogenesis caused by RIO kinase loss. However, the RIO kinases may have a much more direct role in promoting and maintaining Akt activity given that RIOK2 binds to RIOK1 and to components of the TORC2 complex, which is consistent with recent studies in yeast showing that Rio2p can bind to Tor2 [22]. Given that Rio2p is released from mature ribosomes in a regulated process [20], it is possible that the reason mature ribosomes promote TORC2 signaling is because they release free cytoplasmic RIOK2 that then stimulates TORC2 assembly or activity. The specific interplay between the RIO kinases and mTor signaling, ribosome biogenesis, protein translation, and Akt signaling will require additional investigation in the context of both normal and abnormal PI3K and RTK signaling, and may involve other as yet undetermined factors.

Although RIOK1 and RIOK2 loss can cause defects in ribosome maturation [17], [18], in GBM cells the effects of RIO loss are not generic and instead are genotype-specific: p53 null mutant GBM cells showed no major cell cycle defects or apoptosis upon loss of these kinases. This specificity is derived from p53 upregulation and activation induced by the RpL11 ribosomal protein in response to RIOK loss. In humans, activation of the RpL11-p53-dependent ribosomal-stress checkpoint is associated with diseases caused by ribosomal protein haploinsufficiency, such as Diamond-Blackfan anemia, which are characterized by stem and progenitor cell failure [52], [53]. Similarly, in *Drosophila*, haploinsufficiency of genes that encode ribosomal proteins retards developmental cell proliferation [62]. Given that cancer cells share many properties with stem and progenitor cells, induction of the RpL11-p53 ribosomal stress checkpoint may prove useful to deplete cancer cells. Indeed, recent experimental evidence indicates that the RpL11-p53-dependent ribosomal stress checkpoint suppresses tumorigenesis in mouse cancer models [63]. Moreover, several chemotherapeutic drugs induce the ribosomal stress checkpoint [64], [65]. However, many of these drugs have deleterious effects unrelated to ribosomal stress, limiting their use. More specific induction of the ribosomal stress checkpoint, perhaps through RIO kinase inhibition, may prove therapeutically useful for GBM as well as related cancers.

The importance of RIO kinases in cancer cell survival has been validated in independent studies. RIOK2 was recently identified in an RNAi-based screen for kinases that are required for survival of glioma stem-like cells, which confirms our results, although the functionality of RIOK2 in glioma was not explored [66]. In addition, RIOK1 was identified in a cell-based RNAi screen for genes required for Ras-mediated cell survival, although the functionality of RIOK1 was not explored in this study [23]. Of note, almost all other published cell culture-based RNAi screens in GBM cell lines did not pick up RIOK1 or RIOK2 because these screens were not designed to distinguish between kinases that were required for genotypic-specific survival or growth of GBM cell lines, and instead focused on kinases that showed a common requirement in all glioblastoma cell lines tested, be they mutant or wild-type for p53, EGFR, or PTEN [67], [68], [69]. Our results, which are derived from independent multidisciplinary assays, are the first to establish functional connections between the RIO kinases, oncogenesis, Akt signaling, and the RpL11-p53-dependent ribosomal stress checkpoint (Figure 7). We envision that RIOK loss-of-function phenotypes in GBM cells are due to the combined effects of Akt inhibition and p53 induction, which together stimulate apoptosis and cell cycle exit of EGFR- and PTEN- mutant GBM cells, which share a common dependence on Akt signaling (Figure 7). Further studies to address the mechanisms by which the RIO kinases modulate Akt and p53 activity may lead to important new insights into the interactions between both of these pathways in both normal and cancer cells.

## Materials and Methods

### Fly stocks, genetics, culture conditions, immunohistochemistry, and gefitinib treatment

Flies were cultured at 25°C unless otherwise noted. Genotypes were established by standard genetics. Larval brain phenotypes were assessed and imaged as previously described [9]. Stocks were obtained from VDRC, NIG, and Bloomington stock centers (Table S1). *wor-Gal4* lines were from C. Doe. To create *UAS-ΔEGFR* constructs, a full-length human ΔEGFR cDNA was cloned into pUAS-T, and fly stocks with stable insertions were created.

The screen was based on crosses (see Text S1 for genetic methodology) that generated progeny containing a single RNAi construct exclusively expressed in GFP-labeled glia along with dEGFRλ and dp110^CAAX^. Transgenes were overexpressed using the glial-specific *repo-Gal4* transcriptional driver. Screening was performed using fluorescence microscopy to visualize GFP-labeled glia in living larvae, and phenotypes were confirmed with confocal microscopy. Each positive-scoring RNAi construct was tested at least twice. Positive scoring RNAi constructs were also tested in wild-type glia, neuroblasts, and neurons (Text S1, Table S4)

### Mammalian tissue culture techniques and RNAi

Established primary neurosphere cultures (gifts of H. Kornblum) were maintained as previously described in DMEM/F12 medium supplemented with bFGF and EGF [35], [41]. Neurosphere cultures of GBM39 and GBM6 were created from serial xenografts of human GBMs (gifts of C.D. James). Cultured normal human glia were derived from a fresh surgical specimen of normal human cortex (gift of J. Olson) procured under a protocol approved by the Emory University institutional review board. Cultured mouse *PTEN^−/−^; Ink4a/arf^−/−^* astrocytes (gift of R. Bachoo) were maintained in DMEM with 10% serum. The RIOK2 cDNA (Origene) was overexpressed in *PTEN−/−; Ink4a/arf−/−* astrocytes from the pBabe retroviral vector.

The following drugs were used: Nutlin-3 (Cayman), MG132, Akt inhibitor IV (Calbiochem), temozolomide (Tocris), doxorubicin, rapamycin, PP242 (Santa Cruz), PI-103, ZVAD (Enzo), A443654 (gift of Greg Riggins), gefitinib (LC Laboratories), BEZ-235 (Biovision), LY294002 (Cell Signaling Technology), MK-2206, and GDC-0941 (Selleck). Doses of LY294002, BEZ-235, PI-103, GDC-0941, and MK-2206 used on GBM cells were determined by using dose response assays to find the concentrations at which cells showed substantial reduction (approximately <20% of normal) in Akt-mediated phosphorylation of PRAS40 (as detected by immunoblot).

Lentiviral shRNA pLKO.1 plasmids were obtained from the Broad Institute of MIT. RIOK1 shRNAs: TRCN0000196278 and TRCN0000196981. RIOK2 shRNAs: TRCN0000197250, TRCN0000196672, and TRCN0000196684. pLKO.1-GFP and a nontargeting shRNA against lacZ (in pLKO.1) were used as controls. Lentivirus was produced and used as per standard protocols (Sigma). Knockdown was evident by western blot 96 hrs post-infection. For neurosphere cultures, lentivirus was prepared in DMEM/F12 without serum, and infections were done on cells were plated adherently [36].

For siRNAs, all constructs were transfected at 50–100 µM with RNAimax (Invitrogen). Unless otherwise noted, siRNA-treated cells were harvested at 72 hrs post-transfection. 2 sets of pooled siRNAs were tested each for RIOK1 and RIOK2 (Dharmacon), and two different nontargeting siRNAs against GFP or luciferase were used as controls (Dharmacon). For knockdown of p53, p53 siRNAs were used and compared to matched control nontargeting siRNAs (Cell Signaling Technologies). Target sequences are listed in Text S1. For dual-knockdown experiments, U87MG cells were preferred because, with the necessary higher doses of siRNAs, U87MG-ΔEGFR cells showed nonspecific alterations in ΔEGFR expression that affected RIOK levels.

### Immunoblot analysis

Cells were lysed in RIPA buffer and cleared lysates were subjected to standard immunobloting. The following primary antibodies were used: RIOK1 (Novus), RIOK2 (Sigma), p53 (Santa Cruz), p21, EGFR (BD), actin (DSHB), RpL11 (Invitrogen), NRBP2 (Abcam), STK17a/DRAK1 (Anaspec), PDGFRα, VRK1, CDK9, CDK7, STK17B/DRAK2, TLK1, phospho-Akt(S473), phospho-Akt(T308), phospho-PRAS40(T246), phospho-FOXO1(T24)/FOXO3(T32), Akt, phospho-NDRG1(T346), NDRG1, PRAS40, phospho-4E-BP1, 4E-BP1, PARP, cleaved caspase, FOXO3, TSC2, phospho-TSC2, mTor, phospho-mTor(T2448) (Cell Signaling Technologies)

### WST1 and FACS assays

For WST-1 assays, cell lines were infected with lentiviral shRNA constructs and placed under selection for 48 hrs. Following selection, cells were plated for WST1 assays for cell proliferation/viability as per manufacturer's instructions (Clontech). For flow cytometry (FACS) analysis of DNA content, cells were dissociated and stained with propidium iodide (PI). For FACS analysis for apoptosis, cells were treated with indicated siRNAs or lentiviral vectors and stained with Annexin V-FITC and PI or 7AAD (Invitrogen, BD Biosciences). Assays were performed on a FACScaliber II flow cytometer and data were collected using FACSdiva software (BD Biosciences). Cell cycle profiles were generated using ModFit LT (Verity Software House). In all cases, at least 5000 cells were analyzed per sample.

### RIOK2 overexpression and immunoprecipitation

293T cells were transiently transfected with Myc-DDK-tagged RIOK2 constructs. Cells were lysed in 50 mM HEPES pH 7.5, 150 mM NaCl, 5 mM EDTA, 1 mM DTT buffer with protease and phosphatase inhibitors [22]. RIOK2 was immunoprecipitated with M2-agarose (Sigma) from cleared lysates, and washed immunoprecipitates were subjected to immunobloting.

### Biopsy lysates and immunohistochemistry on tumor samples

Human brain tumor biopsies and tissues were obtained from the Brain Tumor Translational Resource under a protocol approved by the University of California, Los Angeles institutional review board. Paraffin embedded human brain tumor specimens and tumor tissue microarrays with matched control tissue were prepared and sectioned using the UCLA Pathology Histology and Tissue Core Facility. Immunohistochemical staining was performed as previously described [70] or as specified by manufacturer guidelines (Sigma). The results were scored by neuropathologists according to standard clinical criteria, and images of RIOK2 immunoreactivity were taken on an Olympus DP72.

### Mouse intracranial graft assays

For orthotopic implantation of mouse astrocytes engineered in vitro, low passage cells (no more than 8–10 passages) were used in two separate experiments. 1×10^5^ cells in 5 µl of PBS were injected stereotactically 2 mm lateral to the midline and 1 mm anterior to the bregma into the brains of 5–6 week old athymic *nu/nu* mice. Mice were monitored and all animals were sacrificed upon evidence of neurological symptoms in experimental groups such that all samples were time-matched. Brains were removed for processing and histological analysis. Sections were scored independently by two neuropathologists for the presence of tumors and injection-associated needle scars. Animals injected with RIOK2-expresing cells that developed tumors outside of the brain (n = 1) were excluded from the final tally. All animal experiments were approved and conducted according to animal welfare guidelines of the IACUC at the University of California, San Diego.

## Supporting Information

Figure S1 ΔEGFR signaling drives glial neoplasia. (A) Optical projections of whole brain-ventral nerve cord complexes from late 3^rd^ instar larvae, approximately 130 hrs old, displayed at the same scale. Genotypes matched to those displayed in close-ups in B–G. Dorsal view; anterior up. Glia are labeled with GFP (green) driven by *repo-Gal4*. (B–G) 3 µm optical projections of brain hemispheres from late 3^rd^ instar larvae, displayed at the same scale. Frontal sections, midway through brains. Anterior up; midline to left. Glial cell nuclei labeled with Repo (red); glial cell bodies labeled with GFP (green). Brains counter-stained with anti-HRP (blue), which reveals neuropil at high intensity and neuronal cell bodies at low intensity. (B, C) glial-specific overexpression of ΔEGFR in the larval brain induced excess glial cell numbers, brain enlargement, and lethality that was rescued with gefitinib (A, F, H). Co-overexpression of ΔEGFR with dp110^CAAX^ produced lethal neoplasia, very similar to that of *dEGFRλ;dp110^CAAX^* animals, that was partially suppressed by gefitinib treatment (A, D, G). However, gefitinib treatment did not fully suppress the growth of neoplastic ΔEGFR; dp110^CAAX^ glia nor did it rescue lethality caused by ΔEGFR; dp110^CAAX^ overexpression (G, H).
(TIF)

Figure S2 Classification of EGFR; PI3K modifiers. (A) Ven diagram comparing confirmed modifiers from this screen to other relevant RNAi-screens in *Drosophila* and to orthologous human kinases implicated in GBM, individual kinases noted in Table S6. (B, C) Network diagrams adapted from STRINGS showing functional connections between *Drosophila* modifier kinases (B), and functional connections between modifier kinases according to orthology information using COG (Clusters of Orthologous Groups) analysis (C) [26]. Confidence views of networks are presented such that stronger associations are represented by thicker lines. Orthologs of kinases implicated in GBM are shown in black, novel modifiers are shown in green (suppressors) and in red (enhancers). Many of the novel modifiers do not have established functional links to RTK or PI3K signaling in *Drosophila* (B). When network analysis takes into account datasets from orthologous kinases in other organisms, such as yeast (C), several of the novel modifiers show connections with each other and with other categorized modifiers of RTK and PI3K signaling, suggesting that these novel modifiers represent new pathway components.
(TIF)

Figure S3 Expression of human orthologs of modifier kinases in high-grade human gliomas. (A–F) Representative immunohistochemical stains for each indicated protein performed on high-grade malignant glioma tumor tissue, all done as part of the Human Protein Atlas Project. CDK7 and CDK9 are nuclear proteins, and show enriched immunoreactivity in tumor cells. TNK2 and RIOK2 are known or predicted cytoplasmic proteins. Antibodies were extensively validated as described in HPA [73], and this data is available at www.proteinatlas.org. Immunostains for each protein were performed on panels of 10–24 tumors, and this data is summarized in Table S10.
(TIF)

Figure S4 Expression of modifier orthologs in cultured GBM cells expressing ΔEGFR. U87MG and U87MG-ΔEGFR cells were cultured with .1% serum for 36 hrs to isolate ΔEGFR signaling, and their extracts were immunobloted for indicated proteins. ΔEGFR runs below full-length EGFR. Proteins that show upregulation in U87MG-ΔEGFR cells are each indicated with arrows.
(TIF)

Figure S5 RIO kinase expression in a panel of GBM cell lines. Indicated cell lines were cultured with .1% serum for 36 hrs to reduce expression artifacts from serum treatment, and their extracts were immunobloted for indicated proteins. PTEN mutant status is shown; SF767 is documented to be PTEN wild-type, while all others have been documented to be PTEN protein null mutant.
(TIF)

Figure S6 p110 and Akt inhibition, but not mTor inhibition, alters RIOK2 expression. (A) U87MG (parent) compared to U87MG-ΔEGFR cells, cultured in .1% serum and treated for 48 hrs with DMSO, 500 nM BEZ-235, or 2 µM PI-103, or treated for 24 hrs with DMSO or 1 µM A443654. PI3K inhibition by BEZ-235 and PI-103 shown by reduced Akt phosphorylation at Serine-473; the blot for Akt-Ser473 phosphorylation has been overexposed to highlight the degree of inhibition of PI3K signaling by BEZ-235 and other compounds rather than the differences in Akt-Ser473 phosphorylation between U87MG and U87MG-ΔEGFR (see Figure 2A). (B) U87MG compared to U87MG-ΔEGFR cells, cultured in .1% serum and treated for 24 hrs with DMSO (both U87MG and U87MG-ΔEGFR), 1 nM rapamycin, or 2 µM PP242, which is an inhibitor of mTor kinase activity. Inhibition of mTor kinase activity is evident by reduced Akt phosphorylation at Serine-473 and/or reduced 4E-BP1 phosphorylation. Increased 4E-BP1 phosphorylation was induced by rapamycin treatment, likely due to positive feedback [74]. RIOK2 is clearly elevated in the presence of EGFR, and is not decreased upon mTor inhibition. RIOK1 shows some reduction with PP242 treatment, but less so with rapamycin treatment.
(TIF)

Figure S7 Akt signaling regulates RIO kinase protein stability. (A) U87MG-ΔEGFR cells were infected with retroviruses containing PTEN, PTEN-G129R (catalytically inactive), or empty vector. Cells were serum-starved for 48 hours and treated for 8 hours with 10 µM MG132, a proteosomal inhibitor. Reduced Akt phosphorylation at Serine-473 is evidence of inhibition by PTEN. (B, C) U87MG-ΔEGFR (B) cells or GBM301 (C) cells were treated with DMSO or 2 µM A443654 with and without 10 µM MG132 for 10 hrs. Akt inhibition is evidenced by reduced PRAS40 phosphorylation in A443664 treated samples.
(TIF)

Figure S8 RIOK2 motif scan. The RIOK2 protein sequence was examined with the Scansite Motif Scanner (http://scansite.mit.edu/motifscan_seq.phtml) [75]. The kinase domain, which is highly similar to that of RIOK1 (RIO1), is indicated in blue. Potential phosphorylation sites are indicated by residue, and lower scores indicate that the predicted site falls into the top percentiles set by high stringency. Serine-483 in RIOK2 has been confirmed as a site of phosphorylation by several unpublished proteomic analyses available at PhosphoSitePlus (http://www.phosphosite.org) [76].
(TIF)

Figure S9 Potential phosphorylation of RIOK2 by Akt. Epitope-tagged RIOK2 (RIOK2-flag) was immunoprecipitated and detected by antibodies specific to Akt substrates phosphorylated on serine or threonine at characteristic Akt target sites (RXXS/T). RIOK2-KD-S483A-flag is an epitope tagged mutant form of RIOK2 which contains a serine-to-alanine change in Serine-483, which is a candidate Akt phosphorylation site (see Figure S8) and two point mutations that render RIOK2 kinase dead to block potential autophosphorylation [18]. Mutation of Serine-483 and the kinase domain did not block the ability of the Akt substrate antibody to detect RIOK2, indicating that other phosphorylation sites in RIOK2 are also recognized by the antibody.
(TIF)

Figure S10 RIOK2 expression in the giant cell and pseudopallisade fractions of GBM tumors. Immunohistochemical staining for RIOK2 (reddish brown), with hematoxilin counterstain. Wider views of heterogeneous RIOK2 immunoreactivity in ΔEGFR-positive human GBMs with giant cell components (A, B), cropped section of A also shown in Figure 4B. Wider view showing RIOK2 immunoreactivity in pseudopallisades (C), also shown cropped in Figure 4D, with high magnification (D) to show enrichment for RIOK2 present in the cellular fraction of pseudopallisades.
(TIF)

Figure S11 Akt and EGFR in GBM tumor tissues positive for RIOK2 expression. Immunohistochemical stains for EGFR and Akt done on sections from the same tumor samples stained for RIOK2 in Figure 4B/Figure S10A (A) and Figure 4E (B). Both tumors show strong expression of EGFR and phosphorylated Akt (Akt-S473-P).
(TIF)

Figure S12 WST1 proliferation assays reveal a requirement for modifier kinases in GBM cells. (A–C) WST-1 assays performed on U87MG cells for indicated genes. Following selection for shRNA expression, proliferation was measured with WST-1 reagent and quantified as the fold increase in absorbance between day 0 and day 3, normalized to controls treated with a nontargeting shRNA. 2–3 shRNAs tested per kinase. P values refer to one-way ANOVA with Dunnett post test.
(TIF)

Figure S13 RIOK knockdown verification for Figure 5. (A, B) Verification of RIOK1 and RIOK2 knockdown in U87MG (A) and U87MG-ΔEGFR (B) cells treated with the indicated shRNAs and subjected to WST1 assays as shown in Figure 5A (left panels). (C) Verification of RIOK2 knockdown in LNZ308 (C) cells treated with the indicated shRNAs and subjected to WST1 assays as shown in Figure 5A (right panel). (D, E) Verification of RIOK1 and RIOK2 knockdown in U87MG (D) and LNZ308 (E) cells treated with the indicated siRNAs and subjected to FACS analysis for cell cycle progression as shown in Figure 5B. (F) Upregulation of p53 upon RIOK2 knockdown with shRNAs. (G) Verification of p53 knockdown in U87MG cells treated with siRNAs against p53 and the RIOKs and subject to FACS analysis of apoptosis as shown in Figure 5E.
(TIF)

Figure S14 Loss of RIOK1 and RIOK2 induces L11-dependent p53 upregulation. RIOK1 or RIOK2 knockdown upregulates p53 and p21 expression in A712 (A) and U178 (B) GBM cells, which is blocked by concurrent RpL11 knockdown. A172 cells are PTEN-mutant and EGFR-mutant, U178 cells are PTEN-mutant, and both are wild-type for p53 [38], [39], [50]. For RpL11 co-knockdown experiments, cells were treated with equivalent amounts of siRNAs mixed 1∶1 for all control and experimental samples. Cells were harvested 72 hrs post-transfection with siRNAs.
(TIF)

Figure S15 RIOK loss chemosensitizes GBM cells. Representative scatter plots of FACS-based quantification of chemosensitivity. U87MG cells were treated with 1 µg/mL doxorubicin and 100 µM temozolomide for 12 hrs beginning 96 hrs post shRNA infection. Treated cells were collected and stained live for 7AAD and Annexin-V. 7AAD alone identifies dead cells (Q1). Annexin V identifies cells in early apoptosis when alone (Q4) and in late stage apoptosis when coincident with 7AAD (Q2). Viable cells (Q3) stained for neither. The percentage of Annexin-V-positive cells present upon doxorubicin and temozolomide treatment significantly increased in RIOK1-shRNA and RIOK2-shRNA treated cells compared to cells treated with a non-targeting control shRNA.
(TIF)

Figure S16 Nutlin-3 treatment does not phenocopy RIOK2 loss. (A) U87MG cells treated with 10 µM nutlin-3 (red) or DMSO (control, black) for 48 hrs prior to fixation and propidium iodide staining for DNA content for cell cycle analysis by FACS. (B) Immunnoblot showing that nutlin-3 treatment significantly increased p53 and p21 levels in U87MG cells, as compared to both control cells and siRNA-RIOK2 treated cells. (C) GBM301 cells plated adherently and infected with a GFP control lentivirus (left panel) or an shRNA lentivirus targeting RIOK2 (right panel). Brightfield images taken 96 hrs post-infection. RIOK2 protein levels drop starting about 72 hrs post-infection, such that 96 hrs is equivalent to 24 hrs of knockdown. Adherent GBM301 cells treated with 10 µM nutlin-3 for 24 hrs (middle panel). Nutlin-3 largely decreased growth of GBM301 cells, whereas RIOK2 knockdown more prominently stimulated apoptosis, yielding many pyknotic and vacuolated cells.
(TIF)

Figure S17 Loss of RIOK1 and RIOK2 function reduces TORC2-Akt signaling. (A) GBM6, a ΔEGFR-positive and p53 mutant neurosphere line. RIOK1 or RIOK2 knockdown caused reduced phosphorylation of Akt on the TORC2 target site, Serine-473, which is clear relative to total Akt protein. Cells harvested 5 days post-infection with viral vectors that target RIOK1/2 or a control vector, treated with 25 µM ZVAD for 48 hrs prior to harvest. (B) RIOK1 knockdown caused reduced phosphorylation of Akt on Serine-473 in U87MG-ΔEGFR cells. Reduced phosphorylation of FOXO3 at the TORC2-dependent Akt target site was detected upon RIOK1 knockdown. Cells harvested 5 days post-infection with viral vectors that target RIOK1/2 or a control vector, treated with 25 µM ZVAD for 48 hrs prior to harvest. (C) RIOK2 knockdown caused reduced phosphorylation of Akt on Serine-473 in U87MG cells. RIOK2 knockdown was also associated with reduced phosphorylation of NDRG1, aTORC2 read-out, and increased p53 protein levels. Cells treated with nontargeting control siRNAs or RIOK2 siRNAs, harvested 96 hrs post-transfection.
(TIF)

Figure S18 Expression of RIOK2 in other tumor types. (A–F) Representative immunohistochemical stains for RIOK2 performed on malignant tumor tissue, all done as part of the Human Protein Atlas Project [73]. (A) breast cancer (duct carcinoma), (B) lung cancer (squamous cell carcinoma), (C) colorectal cancer (adenocarcinoma), (D) head and neck cancer (squamous cell carcinoma), (E) prostate cancer (adenocarcinoma), and (F) malignant melanoma. RIOK2 showed strong expression in 92–100% of colon and head and neck tumors examined, in 50–58% of lung, prostate, and melanoma tumors examined, and in 25% of breast tumors examined (complete analysis and images available at www.proteinatlas.org).
(TIF)

Table S1 All kinases in the *Drosophila* genome. Kinases that are confirmed hits in the dEGFRλ;dp110^CAAX^ screen are in bold, kinases not tested due to lack of available constructs are in grey italics.
(XLS)

Table S2 Screen results from all UAS-dsRNA stocks tested. VDRC stock ID numbers prefaced by “v,” Blooming stock ID numbers prefaced by “b,” NIG stock ID numbers prefaced by an “n,” TRIP stock numbers prefaced with a “t.” Bold highlights all stocks that yielded reproducible genetic interactions and clear phenotypic alterations. Genes that are considered confirmed hits have multiple *UAS-dsRNA* stocks listed in bold and are highlighted in yellow. Key to genetic interactions: N: no interaction/no difference, WS: weak suppressor, S: moderate suppressor, SS: strong suppressor, WE: weak enhancer, E: moderate enhancer, SE: strong enhancer. “?” denotes interactions that were milder than the “weak” designation but subtly different from controls.
(XLS)

Table S3 Dominant negative constructs, overexpression constructs, and mutant alleles tested. Blooming stock ID numbers prefaced by “b.” Bold highlights all confirmed stocks that yielded reproducible genetic interactions and clear phenotypic alterations. Key to genetic interactions: N: no interaction/no difference, WS: weak suppressor, S: moderate suppressor, SS: strong suppressor, WE: weak enhancer, E: moderate enhancer, SE: strong enhancer. “?” denotes interactions that were milder than the “weak” designation but subtly different from controls.
(XLS)

Table S4 Testing modifier kinase RNAi constructs in wild-type glia, neuroblasts, neurons, and eye epithelia. VDRC stock ID numbers prefaced by “v,” Blooming stock ID numbers prefaced by “b,” NIG stock ID numbers prefaced by “n.” Not all stocks tested in all assays. Bold highlights all confirmed dEGFRλ; dp110^CAAX^ modifier stocks that yielded reproducible genetic interactions. Key to dEGFRλ; dp110^CAAX^ genetic interactions: N: no interaction, WS: weak suppressor, S: moderate suppressor, SS: strong suppressor, WE: weak enhancer, E: moderate enhancer, SE: strong enhancer. “?” denotes interactions that were marginal. Testing in wild-type neuroblasts and neurons (2× *wor-Gal4*, *elav-Gal4*) was done with *UAS-dcr* in the background to potentiate RNAi. Data from RNAi constructs tested in neuroblasts with *insc-Gal4* derived from http://neuroblasts.imba.oeaw.ac.at 1. “Lethal,” “semi-lethal,” and “viable” refer to whole-animals effects of modifier constructs in an otherwise wild-type background. In glial-specific assays, whole animal lethality indicates that the genes in question may be essential for some aspect of normal glial function or development, but not necessarily for glial proliferation as blocking glial proliferation is not lethal. For analysis of larval glia, fixed specimens were stained for glial cell nuclei/numbers and viewed at higher magnification.
(XLS)

Table S5 *ΔEGFR* and *ΔEGFR;dp110^CAAX^* modifiers, listed by stocks tested. VDRC stock ID numbers prefaced by “v.” Bold highlights all confirmed stocks that yielded reproducible genetic interactions and clear phenotypic alterations. Genes that are considered ‘confirmed hits’ in the initial screen are listed in bold. Key to genetic interactions: N: no interaction/no difference, WS: weak suppressor, S: moderate suppressor, SS: strong suppressor, WE: weak enhancer, E: moderate enhancer, SE: strong enhancer.
(XLSX)

Table S6 Human orthologs and functional classification of confirmed dEGFRλ;dp110^CAAX^ modifier kinases. Orthologs were curated from http://kinase.com/ and names for each were updated according to current Gene ID numbers and NCBI annotations. GO terms from GOEast, http://omicslab.genetics.ac.cn/GOEAST/.
(XLS)

Table S7 Overlap with related RNAi screens in *Drosophila* and human systems. Novel modifier genes not previously implicated in glioblastoma are highlighted in green (suppressors) and red (enhancers). *Drosophila genes that did not emerge from the annotated RNAi screens, but that have well established roles in RTK and PI3K signaling and/or cell proliferation and survival *in vivo*.
(XLS)

Table S8 Human Orthologs for novel modifiers of dEGFRλ;dp110^CAAX^. Orthologs were curated from http://kinase.com/ and names for each were updated to current NCBI annotations. Human kinases selected for study in human GBM model systems are in bold, as are their *Drosophila* counterparts.
(XLS)

Table S9 TCGA Microarray data for human orthologs of novel modifiers. Each value is AgilentG4502A_07 log2 tumor/normal ratio taken from TCGA profiling, found at http://tcga-portal.nci.nih.gov/tcga-portal/AnomalySearch.jsp. Significant overexpression of 3-fold or more (>1.5) is noted in pink. Significantly decreased expression is noted in blue. Average expression for each gene for all samples is noted at the base of the table. Average expression with standard deviation for each gene for EGFR-overexpressors is noted at the base of the table.
(XLSX)

Table S10 Summary of Human Protein Atlas data adapted from www.proteinatlas.org. Genes highlighted in italics show notable upregulation in the indicated cells and tissues, as assessed by IHC.
(XLSX)

Text S1 Supplemental Results and Materials and Methods. Additional detail on validation of novel kinases in mammalian systems, screening methodology, and shRNA and siRNA sequences.
(DOC)

## References

[1] Furnari FB, Fenton T, Bachoo RM, Mukasa A, Stommel JM, et al (2007) Malignant astrocytic glioma: genetics, biology, and paths to treatment. Genes Dev 21: 2683–2710. 17974913 10.1101/gad.1596707

[2] Nishikawa R, Ji XD, Harmon RC, Lazar CS, Gill GN, et al (1994) A mutant epidermal growth factor receptor common in human glioma confers enhanced tumorigenicity. Proc Natl Acad Sci U S A 91: 7727–7731. 8052651 10.1073/pnas.91.16.7727 PMC44475

[3] Mukasa A, Wykosky J, Ligon KL, Chin L, Cavenee WK, et al (2010) Mutant EGFR is required for maintenance of glioma growth in vivo, and its ablation leads to escape from receptor dependence. Proc Natl Acad Sci U S A 107: 2616–2621. 20133782 10.1073/pnas.0914356107 PMC2823874

[4] Holland EC, Celestino J, Dai C, Schaefer L, Sawaya RE, et al (2000) Combined activation of Ras and Akt in neural progenitors induces glioblastoma formation in mice. Nat Genet 25: 55–57. 10802656 10.1038/75596

[5] Marumoto T, Tashiro A, Friedmann-Morvinski D, Scadeng M, Soda Y, et al (2009) Development of a novel mouse glioma model using lentiviral vectors. Nat Med 15: 110–116. 19122659 10.1038/nm.1863 PMC2671237

[6] Bachoo RM, Maher EA, Ligon KL, Sharpless NE, Chan SS, et al (2002) Epidermal growth factor receptor and Ink4a/Arf: convergent mechanisms governing terminal differentiation and transformation along the neural stem cell to astrocyte axis. Cancer Cell 1: 269–277. 12086863 10.1016/s1535-6108(02)00046-6

[7] Holland EC, Hively WP, DePinho RA, Varmus HE (1998) A constitutively active epidermal growth factor receptor cooperates with disruption of G1 cell-cycle arrest pathways to induce glioma-like lesions in mice. Genes Dev 12: 3675–3685. 9851974 10.1101/gad.12.23.3675 PMC317252

[8] Cloughesy TF, Yoshimoto K, Nghiemphu P, Brown K, Dang J, et al (2008) Antitumor activity of rapamycin in a Phase I trial for patients with recurrent PTEN-deficient glioblastoma. PLoS Med 5: e8 doi:10.1371/journal.pmed.0050008. 18215105 10.1371/journal.pmed.0050008 PMC2211560

[9] Read RD, Cavenee WK, Furnari FB, Thomas JB (2009) A Drosophila model for EGFR-Ras and PI3K-dependent human glioma. PLoS Genet 5: e1000374 doi:10.1371/journal.pgen.1000374. 19214224 10.1371/journal.pgen.1000374 PMC2636203

[10] Reiter LT, Bier E (2002) Using Drosophila melanogaster to uncover human disease gene function and potential drug target proteins. Expert Opin Ther Targets 6: 387–399. 12223075 10.1517/14728222.6.3.387

[11] St Johnston D (2002) The art and design of genetic screens: Drosophila melanogaster. Nat Rev Genet 3: 176–188. 11972155 10.1038/nrg751

[12] Dietzl G, Chen D, Schnorrer F, Su KC, Barinova Y, et al (2007) A genome-wide transgenic RNAi library for conditional gene inactivation in Drosophila. Nature 448: 151–156. 17625558 10.1038/nature05954

[13] Bellen HJ, Tong C, Tsuda H (2011) 100 years of Drosophila research and its impact on vertebrate neuroscience: a history lesson for the future. Nat Rev Neurosci 11: 514–522. 10.1038/nrn2839 PMC4022039 20383202

[14] Freeman MR, Doherty J (2006) Glial cell biology in Drosophila and vertebrates. Trends Neurosci 29: 82–90. 16377000 10.1016/j.tins.2005.12.002

[15] LaRonde-LeBlanc N, Wlodawer A (2005) A family portrait of the RIO kinases. J Biol Chem 280: 37297–37300. 16183636 10.1074/jbc.R500013200

[16] Vanrobays E, Gelugne JP, Gleizes PE, Caizergues-Ferrer M (2003) Late cytoplasmic maturation of the small ribosomal subunit requires RIO proteins in Saccharomyces cerevisiae. Mol Cell Biol 23: 2083–2095. 12612080 10.1128/MCB.23.6.2083-2095.2003 PMC149469

[17] Widmann B, Wandrey F, Badertscher L, Wyler E, Pfannstiel J, et al (2011) The kinase activity of human Rio1 is required for final steps of cytoplasmic maturation of 40S subunits. Mol Biol Cell 10.1091/mbc.E11-07-0639 PMC3248900 22072790

[18] Zemp I, Wild T, O'Donohue MF, Wandrey F, Widmann B, et al (2009) Distinct cytoplasmic maturation steps of 40S ribosomal subunit precursors require hRio2. J Cell Biol 185: 1167–1180. 19564402 10.1083/jcb.200904048 PMC2712965

[19] Baumas K, Soudet J, Caizergues-Ferrer M, Faubladier M, Henry Y, et al (2012) Human RioK3 is a novel component of cytoplasmic pre-40S pre-ribosomal particles. RNA Biol 9: 162–174. 22418843 10.4161/rna.18810 PMC3346313

[20] Strunk BS, Novak MN, Young CL, Karbstein K (2012) A translation-like cycle is a quality control checkpoint for maturing 40S ribosome subunits. Cell 150: 111–121. 22770215 10.1016/j.cell.2012.04.044 PMC3615461

[21] Olsen JV, Blagoev B, Gnad F, Macek B, Kumar C, et al (2006) Global, in vivo, and site-specific phosphorylation dynamics in signaling networks. Cell 127: 635–648. 17081983 10.1016/j.cell.2006.09.026

[22] Breitkreutz A, Choi H, Sharom JR, Boucher L, Neduva V, et al (2010) A global protein kinase and phosphatase interaction network in yeast. Science 328: 1043–1046. 20489023 10.1126/science.1176495 PMC3983991

[23] Luo J, Emanuele MJ, Li D, Creighton CJ, Schlabach MR, et al (2009) A genome-wide RNAi screen identifies multiple synthetic lethal interactions with the Ras oncogene. Cell 137: 835–848. 19490893 10.1016/j.cell.2009.05.006 PMC2768667

[24] Manning G, Plowman GD, Hunter T, Sudarsanam S (2002) Evolution of protein kinase signaling from yeast to man. Trends Biochem Sci 27: 514–520. 12368087 10.1016/s0968-0004(02)02179-5

[25] Morrison DK, Murakami MS, Cleghon V (2000) Protein kinases and phosphatases in the Drosophila genome. J Cell Biol 150: F57–62. 10908587 10.1083/jcb.150.2.f57 PMC2180215

[26] Szklarczyk D, Franceschini A, Kuhn M, Simonovic M, Roth A, et al (2011) The STRING database in 2011: functional interaction networks of proteins, globally integrated and scored. Nucleic Acids Res 39: D561–568. 21045058 10.1093/nar/gkq973 PMC3013807

[27] Bjorklund M, Taipale M, Varjosalo M, Saharinen J, Lahdenpera J, et al (2006) Identification of pathways regulating cell size and cell-cycle progression by RNAi. Nature 439: 1009–1013. 16496002 10.1038/nature04469

[28] Bettencourt-Dias M, Giet R, Sinka R, Mazumdar A, Lock WG, et al (2004) Genome-wide survey of protein kinases required for cell cycle progression. Nature 432: 980–987. 15616552 10.1038/nature03160

[29] Friedman A, Perrimon N (2006) A functional RNAi screen for regulators of receptor tyrosine kinase and ERK signalling. Nature 444: 230–234. 17086199 10.1038/nature05280

[30] Boutros M, Kiger AA, Armknecht S, Kerr K, Hild M, et al (2004) Genome-wide RNAi analysis of growth and viability in Drosophila cells. Science 303: 832–835. 14764878 10.1126/science.1091266

[31] Neumuller RA, Richter C, Fischer A, Novatchkova M, Neumuller KG, et al (2011) Genome-Wide Analysis of Self-Renewal in Drosophila Neural Stem Cells by Transgenic RNAi. Cell Stem Cell 8: 580–593. 21549331 10.1016/j.stem.2011.02.022 PMC3093620

[32] Sousa-Nunes R, Yee LL, Gould AP (2011) Fat cells reactivate quiescent neuroblasts via TOR and glial insulin relays in Drosophila. Nature 471: 508–512. 21346761 10.1038/nature09867 PMC3146047

[33] Reddy BV, Irvine KD (2011) Regulation of Drosophila glial cell proliferation by Merlin-Hippo signaling. Development 138: 5201–5212. 22069188 10.1242/dev.069385 PMC3210499

[34] Huang HS, Nagane M, Klingbeil CK, Lin H, Nishikawa R, et al (1997) The enhanced tumorigenic activity of a mutant epidermal growth factor receptor common in human cancers is mediated by threshold levels of constitutive tyrosine phosphorylation and unattenuated signaling. J Biol Chem 272: 2927–2935. 9006938 10.1074/jbc.272.5.2927

[35] Laks DR, Masterman-Smith M, Visnyei K, Angenieux B, Orozco NM, et al (2009) Neurosphere formation is an independent predictor of clinical outcome in malignant glioma. Stem Cells 27: 980–987. 19353526 10.1002/stem.15 PMC3177534

[36] Pollard SM, Yoshikawa K, Clarke ID, Danovi D, Stricker S, et al (2009) Glioma stem cell lines expanded in adherent culture have tumor-specific phenotypes and are suitable for chemical and genetic screens. Cell Stem Cell 4: 568–580. 19497285 10.1016/j.stem.2009.03.014

[37] Lee J, Kotliarova S, Kotliarov Y, Li A, Su Q, et al (2006) Tumor stem cells derived from glioblastomas cultured in bFGF and EGF more closely mirror the phenotype and genotype of primary tumors than do serum-cultured cell lines. Cancer Cell 9: 391–403. 16697959 10.1016/j.ccr.2006.03.030

[38] Ishii N, Maier D, Merlo A, Tada M, Sawamura Y, et al (1999) Frequent co-alterations of TP53, p16/CDKN2A, p14ARF, PTEN tumor suppressor genes in human glioma cell lines. Brain Pathol 9: 469–479. 10416987 10.1111/j.1750-3639.1999.tb00536.x PMC8098486

[39] Ciesielski MJ, Fenstermaker RA (2000) Oncogenic epidermal growth factor receptor mutants with tandem duplication: gene structure and effects on receptor function. Oncogene 19: 810–820. 10698499 10.1038/sj.onc.1203409

[40] Gallia GL, Tyler BM, Hann CL, Siu IM, Giranda VL, et al (2009) Inhibition of Akt inhibits growth of glioblastoma and glioblastoma stem-like cells. Mol Cancer Ther 8: 386–393. 19208828 10.1158/1535-7163.MCT-08-0680 PMC4498795

[41] Pandita A, Aldape KD, Zadeh G, Guha A, James CD (2004) Contrasting in vivo and in vitro fates of glioblastoma cell subpopulations with amplified EGFR. Gene Chromosome Canc 39: 29–36. 10.1002/gcc.10300 14603439

[42] Inda MD, Bonavia R, Mukasa A, Narita Y, Sah DW, et al (2010) Tumor heterogeneity is an active process maintained by a mutant EGFR-induced cytokine circuit in glioblastoma. Genes Dev 24: 1731–1745. 20713517 10.1101/gad.1890510 PMC2922502

[43] Szerlip NJ, Pedraza A, Chakravarty D, Azim M, McGuire J, et al (2012) Intratumoral heterogeneity of receptor tyrosine kinases EGFR and PDGFRA amplification in glioblastoma defines subpopulations with distinct growth factor response. Proc Natl Acad Sci U S A 109: 3041–3046. 22323597 10.1073/pnas.1114033109 PMC3286976

[44] Chen AJ, Paik JH, Zhang H, Shukla SA, Mortensen R, et al (2012) STAR RNA-binding protein Quaking suppresses cancer via stabilization of specific miRNA. Genes Dev 26: 1459–1472. 22751500 10.1101/gad.189001.112 PMC3403014

[45] Ying H, Zheng H, Scott K, Wiedemeyer R, Yan H, et al (2010) Mig-6 controls EGFR trafficking and suppresses gliomagenesis. Proc Natl Acad Sci U S A 107: 6912–6917. 20351267 10.1073/pnas.0914930107 PMC2872443

[46] Guertin DA, Stevens DM, Thoreen CC, Burds AA, Kalaany NY, et al (2006) Ablation in mice of the mTORC components raptor, rictor, or mLST8 reveals that mTORC2 is required for signaling to Akt-FOXO and PKCalpha, but not S6K1. Dev Cell 11: 859–871. 17141160 10.1016/j.devcel.2006.10.007

[47] Sunayama J, Sato A, Matsuda K, Tachibana K, Watanabe E, et al (2011) FoxO3a functions as a key integrator of cellular signals that control glioblastoma stem-like cell differentiation and tumorigenicity. Stem Cells 29: 1327–1337. 21793107 10.1002/stem.696

[48] Tanaka K, Babic I, Nathanson D, Akhavan D, Guo D, et al (2011) Oncogenic EGFR signaling activates an mTORC2-NF-kappaB pathway that promotes chemotherapy resistance. Cancer Discov 1: 524–538. 22145100 10.1158/2159-8290.CD-11-0124 PMC3229221

[49] Bashir T, Cloninger C, Artinian N, Anderson L, Bernath A, et al (2012) Conditional astroglial rictor overexpression induces malignant glioma in mice. PLoS ONE 7: e47741 doi:10.1371/journal.pone.0047741. 23077666 10.1371/journal.pone.0047741 PMC3471885

[50] Furnari FB, Lin H, Huang HS, Cavenee WK (1997) Growth suppression of glioma cells by PTEN requires a functional phosphatase catalytic domain. Proc Natl Acad Sci U S A 94: 12479–12484. 9356475 10.1073/pnas.94.23.12479 PMC25009

[51] Vanrobays E, Gleizes PE, Bousquet-Antonelli C, Noaillac-Depeyre J, Caizergues-Ferrer M, et al (2001) Processing of 20S pre-rRNA to 18S ribosomal RNA in yeast requires Rrp10p, an essential non-ribosomal cytoplasmic protein. EMBO J 20: 4204–4213. 11483523 10.1093/emboj/20.15.4204 PMC149176

[52] Fumagalli S, Thomas G (2011) The role of p53 in ribosomopathies. Semin Hematol 48: 97–105. 21435506 10.1053/j.seminhematol.2011.02.004

[53] Narla A, Ebert BL (2010) Ribosomopathies: human disorders of ribosome dysfunction. Blood 115: 3196–3205. 20194897 10.1182/blood-2009-10-178129 PMC2858486

[54] Simmons ML, Lamborn KR, Takahashi M, Chen P, Israel MA, et al (2001) Analysis of complex relationships between age, p53, epidermal growth factor receptor, and survival in glioblastoma patients. Cancer Res 61: 1122–1128. 11221842

[55] McLendon R, Friedman A, Bigner D, Van Meir EG, Brat DJ, et al (2008) Comprehensive genomic characterization defines human glioblastoma genes and core pathways. Nature 455: 1061–1068. 18772890 10.1038/nature07385 PMC2671642

[56] Ohgaki H, Dessen P, Jourde B, Horstmann S, Nishikawa T, et al (2004) Genetic pathways to glioblastoma: a population-based study. Cancer Res 64: 6892–6899. 15466178 10.1158/0008-5472.CAN-04-1337

[57] Lowe SW, Ruley HE, Jacks T, Housman DE (1993) p53-dependent apoptosis modulates the cytotoxicity of anticancer agents. Cell 74: 957–967. 8402885 10.1016/0092-8674(93)90719-7

[58] Stupp R, Mason WP, van den Bent MJ, Weller M, Fisher B, et al (2005) Radiotherapy plus concomitant and adjuvant temozolomide for glioblastoma. N Engl J Med 352: 987–996. 15758009 10.1056/NEJMoa043330

[59] Villalonga-Planells R, Coll-Mulet L, Martinez-Soler F, Castano E, Acebes JJ, et al (2011) Activation of p53 by nutlin-3a induces apoptosis and cellular senescence in human glioblastoma multiforme. PLoS ONE 6: e18588 doi:10.1371/journal.pone.0018588. 21483692 10.1371/journal.pone.0018588 PMC3071734

[60] Zinzalla V, Stracka D, Oppliger W, Hall MN (2011) Activation of mTORC2 by association with the ribosome. Cell 144: 757–768. 21376236 10.1016/j.cell.2011.02.014

[61] Liu T, Deng M, Li J, Tong X, Wei Q, et al (2011) Phosphorylation of right open reading frame 2 (Rio2) protein kinase by polo-like kinase 1 regulates mitotic progression. J Biol Chem 286: 36352–36360. 21880710 10.1074/jbc.M111.250175 PMC3196107

[62] Marygold SJ, Roote J, Reuter G, Lambertsson A, Ashburner M, et al (2007) The ribosomal protein genes and Minute loci of Drosophila melanogaster. Genome Biol 8: R216. 17927810 10.1186/gb-2007-8-10-r216 PMC2246290

[63] Macias E, Jin A, Deisenroth C, Bhat K, Mao H, et al (2010) An ARF-independent c-MYC-activated tumor suppression pathway mediated by ribosomal protein-Mdm2 Interaction. Cancer Cell 18: 231–243. 20832751 10.1016/j.ccr.2010.08.007 PMC4400806

[64] Burger K, Muhl B, Harasim T, Rohrmoser M, Malamoussi A, et al (2010) Chemotherapeutic drugs inhibit ribosome biogenesis at various levels. J Biol Chem 285: 12416–12425. 20159984 10.1074/jbc.M109.074211 PMC2852979

[65] Sun XX, Dai MS, Lu H (2007) 5-fluorouracil activation of p53 involves an MDM2-ribosomal protein interaction. J Biol Chem 282: 8052–8059. 17242401 10.1074/jbc.M610621200

[66] Goidts V, Bageritz J, Puccio L, Nakata S, Zapatka M, et al (2012) RNAi screening in glioma stem-like cells identifies PFKFB4 as a key molecule important for cancer cell survival. Oncogene 31: 3235–3243. 22056879 10.1038/onc.2011.490

[67] Wurdak H, Zhu S, Romero A, Lorger M, Watson J, et al (2010) An RNAi screen identifies TRRAP as a regulator of brain tumor-initiating cell differentiation. Cell Stem Cell 6: 37–47. 20085741 10.1016/j.stem.2009.11.002

[68] Wiedemeyer WR, Dunn IF, Quayle SN, Zhang J, Chheda MG, et al (2010) Pattern of retinoblastoma pathway inactivation dictates response to CDK4/6 inhibition in GBM. Proc Natl Acad Sci U S A 107: 11501–11506. 20534551 10.1073/pnas.1001613107 PMC2895056

[69] Kim YW, Liu TJ, Koul D, Tiao N, Feroze AH, et al (2011) Identification of novel synergistic targets for rational drug combinations with PI3 kinase inhibitors using siRNA synthetic lethality screening against GBM. Neuro Oncol 13: 367–375. 21430111 10.1093/neuonc/nor012 PMC3064700

[70] Mellinghoff IK, Wang MY, Vivanco I, Haas-Kogan DA, Zhu S, et al (2005) Molecular determinants of the response of glioblastomas to EGFR kinase inhibitors. N Engl J Med 353: 2012–2024. 16282176 10.1056/NEJMoa051918

[71] Raynaud FI, Eccles SA, Patel S, Alix S, Box G, et al (2009) Biological properties of potent inhibitors of class I phosphatidylinositide 3-kinases: from PI-103 through PI-540, PI-620 to the oral agent GDC-0941. Mol Cancer Ther 8: 1725–1738. 19584227 10.1158/1535-7163.MCT-08-1200 PMC2718129

[72] Hirai H, Sootome H, Nakatsuru Y, Miyama K, Taguchi S, et al (2010) MK-2206, an allosteric Akt inhibitor, enhances antitumor efficacy by standard chemotherapeutic agents or molecular targeted drugs in vitro and in vivo. Mol Cancer Ther 9: 1956–1967. 20571069 10.1158/1535-7163.MCT-09-1012

[73] Uhlen M, Oksvold P, Fagerberg L, Lundberg E, Jonasson K, et al (2010) Towards a knowledge-based Human Protein Atlas. Nat Biotechnol 28: 1248–1250. 21139605 10.1038/nbt1210-1248

[74] Choo AY, Yoon SO, Kim SG, Roux PP, Blenis J (2008) Rapamycin differentially inhibits S6Ks and 4E-BP1 to mediate cell-type-specific repression of mRNA translation. Proc Natl Acad Sci U S A 105: 17414–17419. 18955708 10.1073/pnas.0809136105 PMC2582304

[75] Obenauer JC, Cantley LC, Yaffe MB (2003) Scansite 2.0: Proteome-wide prediction of cell signaling interactions using short sequence motifs. Nucleic Acids Res 31: 3635–3641. 12824383 10.1093/nar/gkg584 PMC168990

[76] Hornbeck PV, Kornhauser JM, Tkachev S, Zhang B, Skrzypek E, et al (2011) PhosphoSitePlus: a comprehensive resource for investigating the structure and function of experimentally determined post-translational modifications in man and mouse. Nucleic Acids Res 40: D261–270. 22135298 10.1093/nar/gkr1122 PMC3245126

