# Supplementary figures and images for: A Kinome-Wide RNAi Screen in Drosophila Glia Reveals That the RIO Kinases Mediate Cell Proliferation and Survival through TORC2-Akt Signaling in Glioblastoma
Source: PLoS Genet. 2013 Feb 14;9(2):e1003253. doi: 10.1371/journal.pgen.1003253 (PMC3573097; doi:10.1371/journal.pgen.1003253)

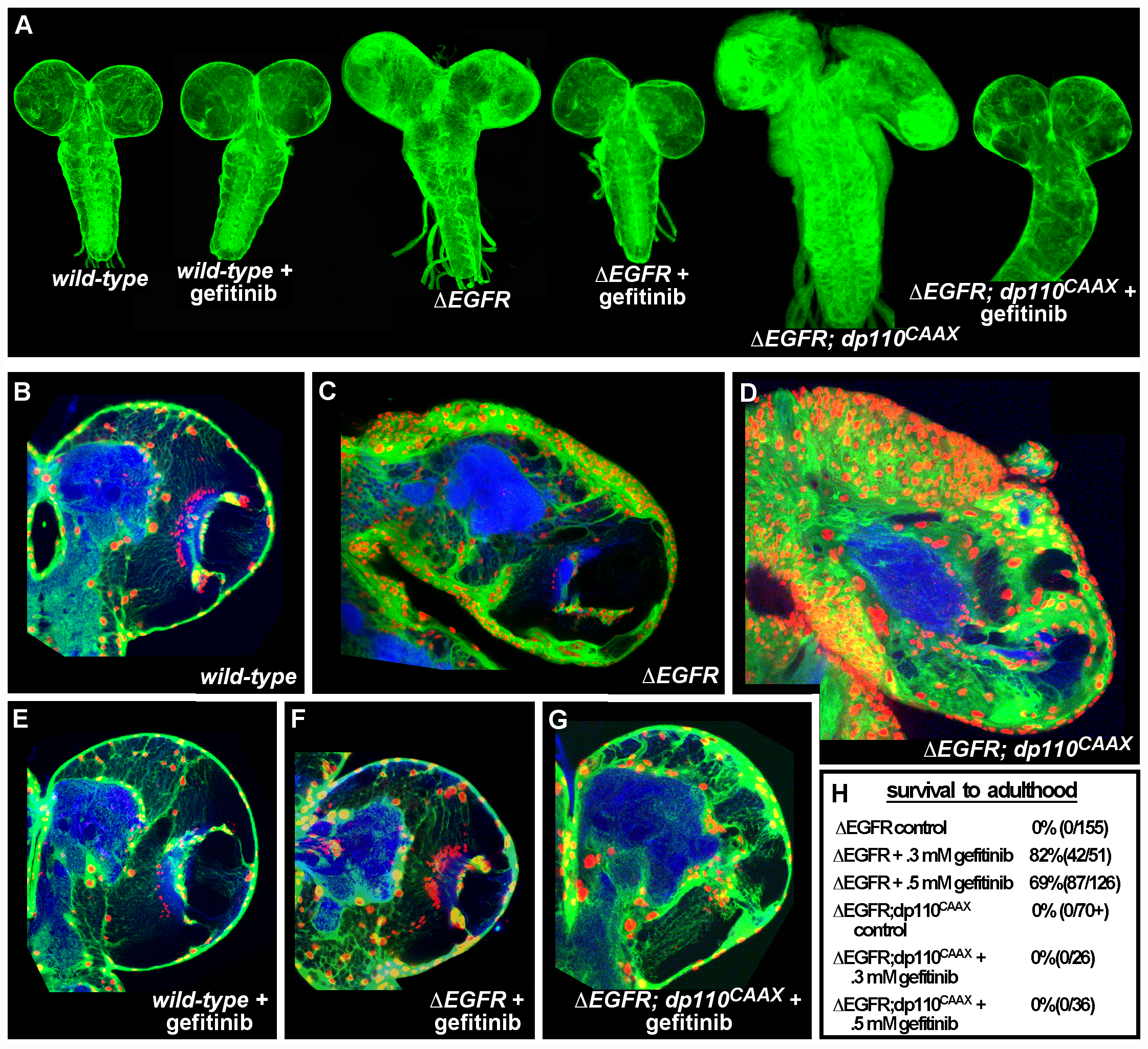

Supplement: Figure S1 — ΔEGFR signaling drives glial neoplasia. (A) Optical projections of whole brain-ventral nerve cord complexes from late 3rd instar larvae, approximately 130 hrs old, displayed at the same scale. Genotypes matched to those displayed in close-ups in B–G. Dorsal view; anterior up. Glia are labeled with GFP (green) driven by repo-Gal4. (B–G) 3 µm optical projections of brain hemispheres from late 3rd instar larvae, displayed at the same scale. Frontal sections, midway through brains. Anterior up; midline to left. Glial cell nuclei labeled with Repo (red); glial cell bodies labeled with GFP (green). Brains counter-stained with anti-HRP (blue), which reveals neuropil at high intensity and neuronal cell bodies at low intensity. (B, C) glial-specific overexpression of ΔEGFR in the larval brain induced excess glial cell numbers, brain enlargement, and lethality that was rescued with gefitinib (A, F, H). Co-overexpression of ΔEGFR with dp110CAAX produced lethal neoplasia, very similar to that of dEGFRλ;dp110CAAX animals, that was partially suppressed by gefitinib treatment (A, D, G). However, gefitinib treatment did not fully suppress the growth of neoplastic ΔEGFR; dp110CAAX glia nor did it rescue lethality caused by ΔEGFR; dp110CAAX overexpression (G, H). (TIF) [file pgen.1003253.s001.tif]

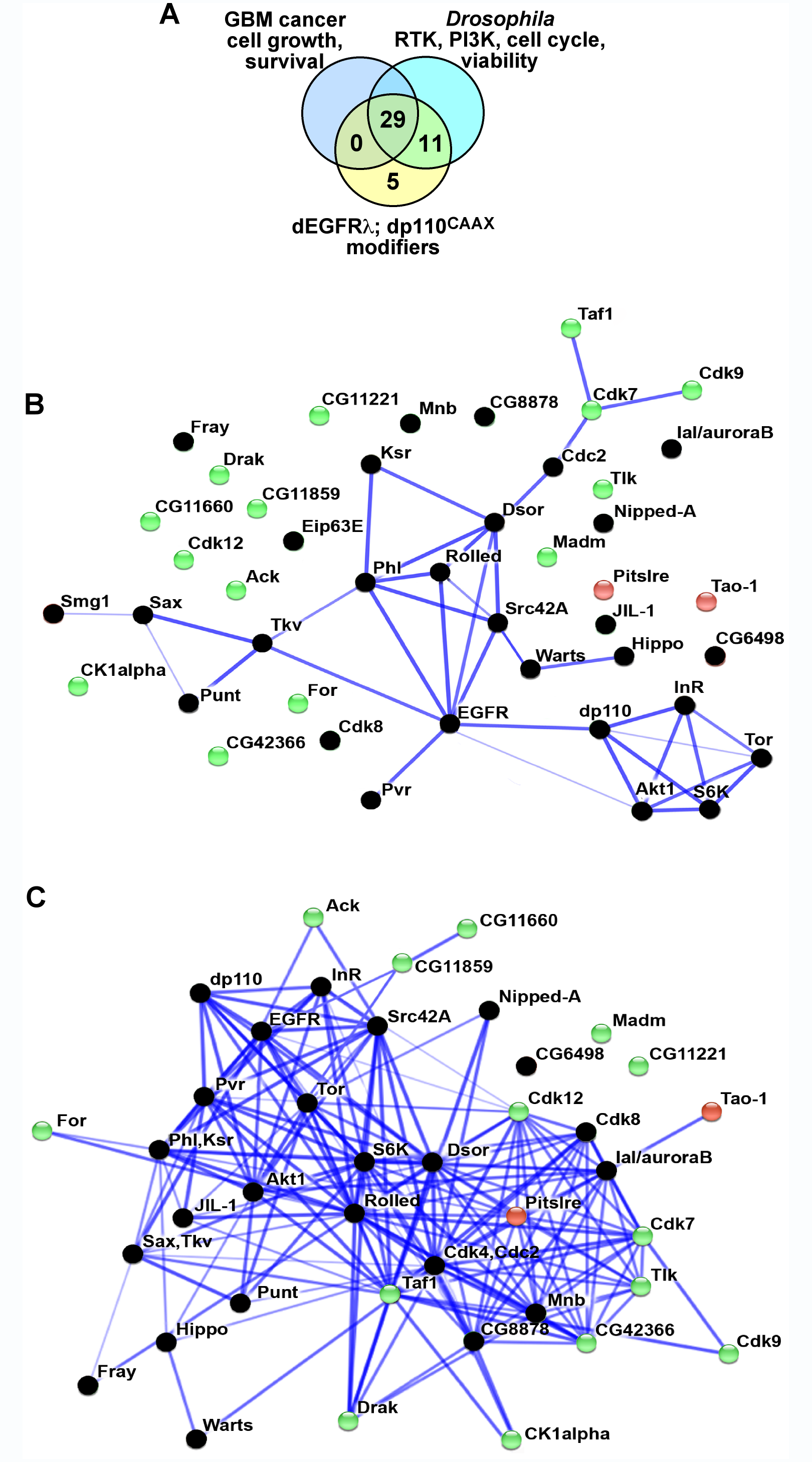

Supplement: Figure S2 — Classification of EGFR; PI3K modifiers. (A) Ven diagram comparing confirmed modifiers from this screen to other relevant RNAi-screens in Drosophila and to orthologous human kinases implicated in GBM, individual kinases noted in Table S6. (B, C) Network diagrams adapted from STRINGS showing functional connections between Drosophila modifier kinases (B), and functional connections between modifier kinases according to orthology information using COG (Clusters of Orthologous Groups) analysis (C) [26]. Confidence views of networks are presented such that stronger associations are represented by thicker lines. Orthologs of kinases implicated in GBM are shown in black, novel modifiers are shown in green (suppressors) and in red (enhancers). Many of the novel modifiers do not have established functional links to RTK or PI3K signaling in Drosophila (B). When network analysis takes into account datasets from orthologous kinases in other organisms, such as yeast (C), several of the novel modifiers show connections with each other and with other categorized modifiers of RTK and PI3K signaling, suggesting that these novel modifiers represent new pathway components. (TIF) [file pgen.1003253.s002.tif]

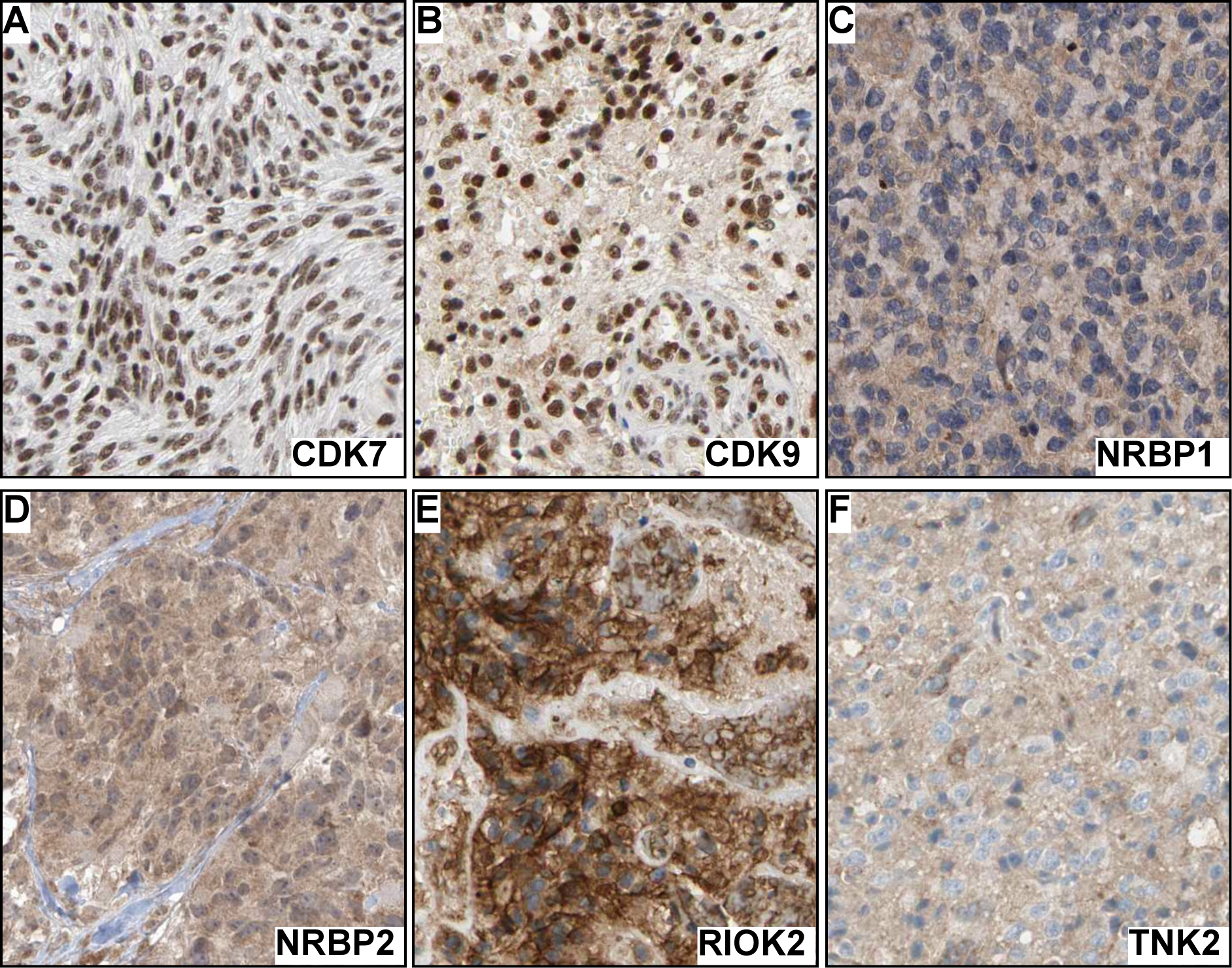

Supplement: Figure S3 — Expression of human orthologs of modifier kinases in high-grade human gliomas. (A–F) Representative immunohistochemical stains for each indicated protein performed on high-grade malignant glioma tumor tissue, all done as part of the Human Protein Atlas Project. CDK7 and CDK9 are nuclear proteins, and show enriched immunoreactivity in tumor cells. TNK2 and RIOK2 are known or predicted cytoplasmic proteins. Antibodies were extensively validated as described in HPA [73], and this data is available at www.proteinatlas.org. Immunostains for each protein were performed on panels of 10–24 tumors, and this data is summarized in Table S10. (TIF) [file pgen.1003253.s003.tif]

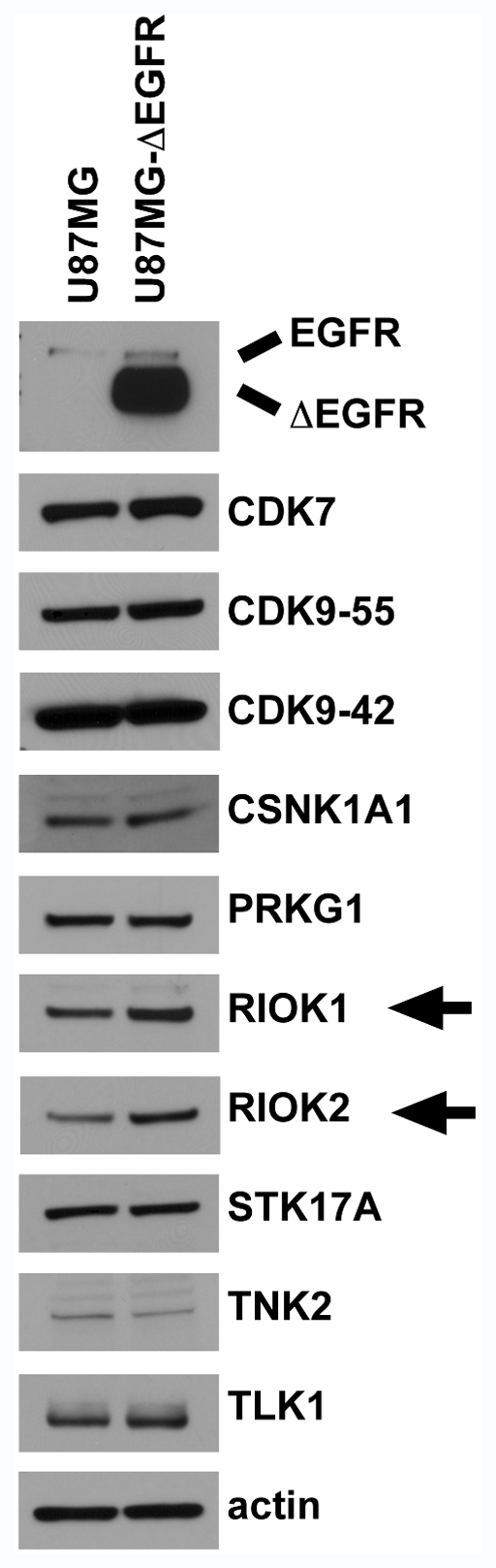

Supplement: Figure S4 — Expression of modifier orthologs in cultured GBM cells expressing ΔEGFR. U87MG and U87MG-ΔEGFR cells were cultured with .1% serum for 36 hrs to isolate ΔEGFR signaling, and their extracts were immunobloted for indicated proteins. ΔEGFR runs below full-length EGFR. Proteins that show upregulation in U87MG-ΔEGFR cells are each indicated with arrows. (TIF) [file pgen.1003253.s004.tif]

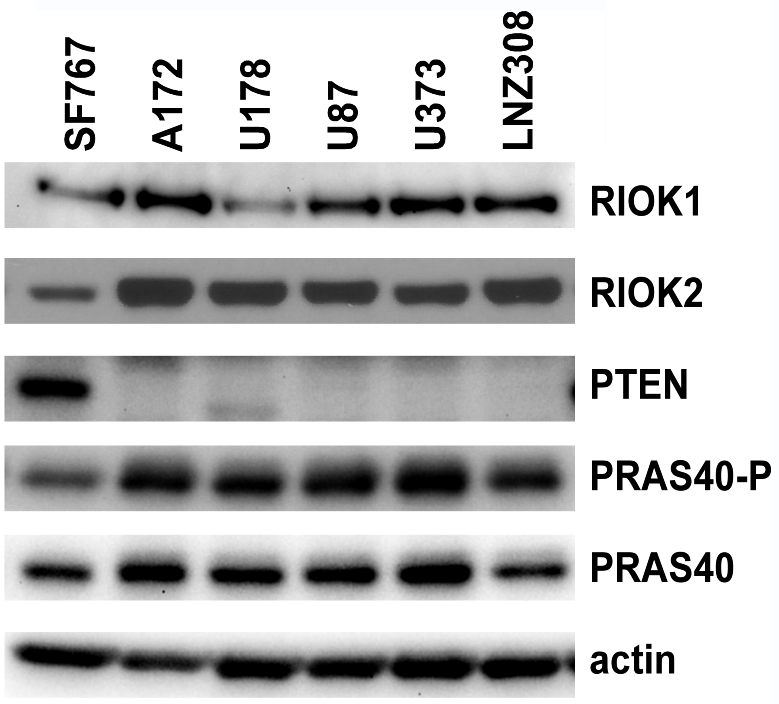

Supplement: Figure S5 — RIO kinase expression in a panel of GBM cell lines. Indicated cell lines were cultured with .1% serum for 36 hrs to reduce expression artifacts from serum treatment, and their extracts were immunobloted for indicated proteins. PTEN mutant status is shown; SF767 is documented to be PTEN wild-type, while all others have been documented to be PTEN protein null mutant. (TIF) [file pgen.1003253.s005.tif]

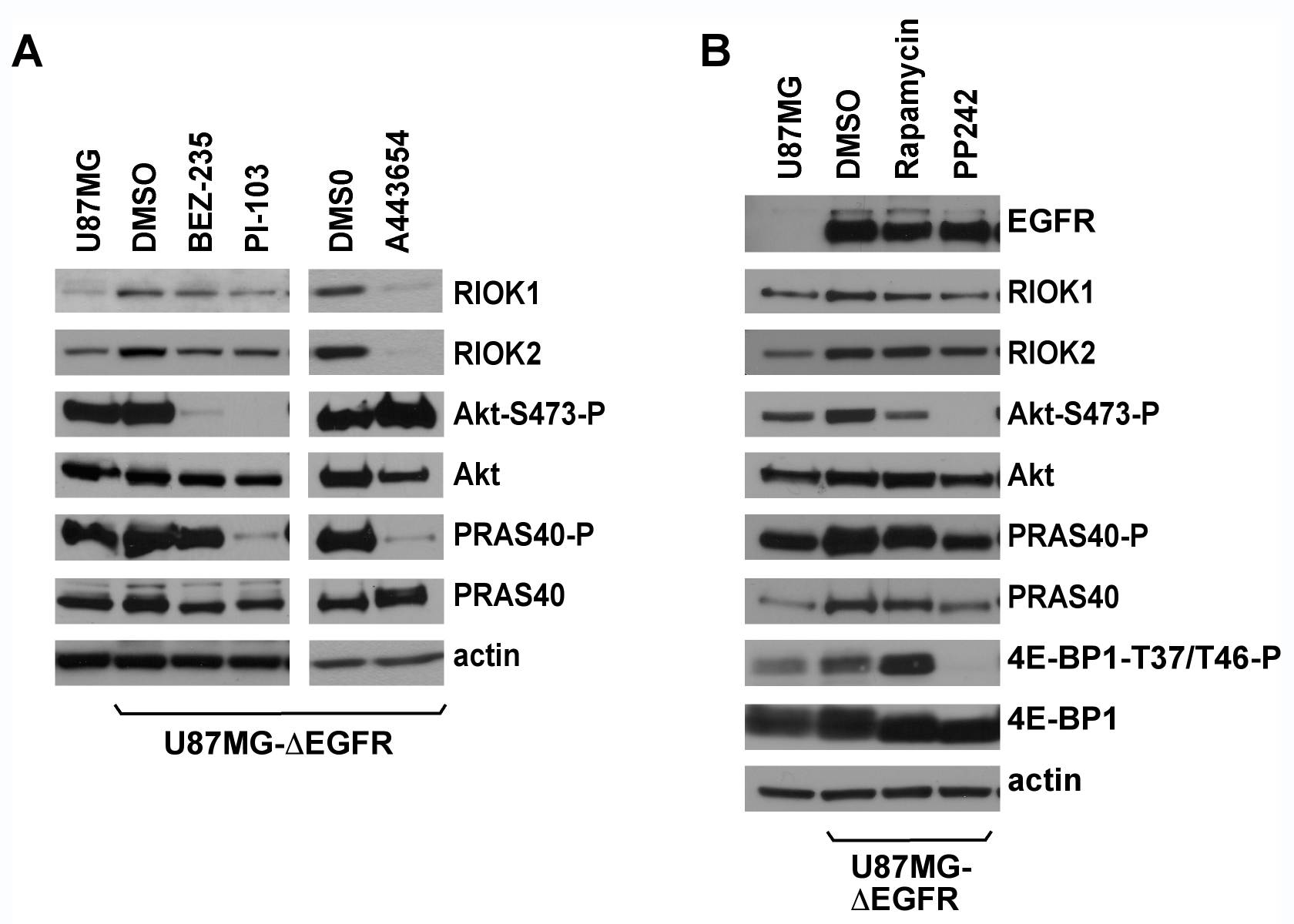

Supplement: Figure S6 — p110 and Akt inhibition, but not mTor inhibition, alters RIOK2 expression. (A) U87MG (parent) compared to U87MG-ΔEGFR cells, cultured in .1% serum and treated for 48 hrs with DMSO, 500 nM BEZ-235, or 2 µM PI-103, or treated for 24 hrs with DMSO or 1 µM A443654. PI3K inhibition by BEZ-235 and PI-103 shown by reduced Akt phosphorylation at Serine-473; the blot for Akt-Ser473 phosphorylation has been overexposed to highlight the degree of inhibition of PI3K signaling by BEZ-235 and other compounds rather than the differences in Akt-Ser473 phosphorylation between U87MG and U87MG-ΔEGFR (see Figure 2A). (B) U87MG compared to U87MG-ΔEGFR cells, cultured in .1% serum and treated for 24 hrs with DMSO (both U87MG and U87MG-ΔEGFR), 1 nM rapamycin, or 2 µM PP242, which is an inhibitor of mTor kinase activity. Inhibition of mTor kinase activity is evident by reduced Akt phosphorylation at Serine-473 and/or reduced 4E-BP1 phosphorylation. Increased 4E-BP1 phosphorylation was induced by rapamycin treatment, likely due to positive feedback [74]. RIOK2 is clearly elevated in the presence of EGFR, and is not decreased upon mTor inhibition. RIOK1 shows some reduction with PP242 treatment, but less so with rapamycin treatment. (TIF) [file pgen.1003253.s006.tif]

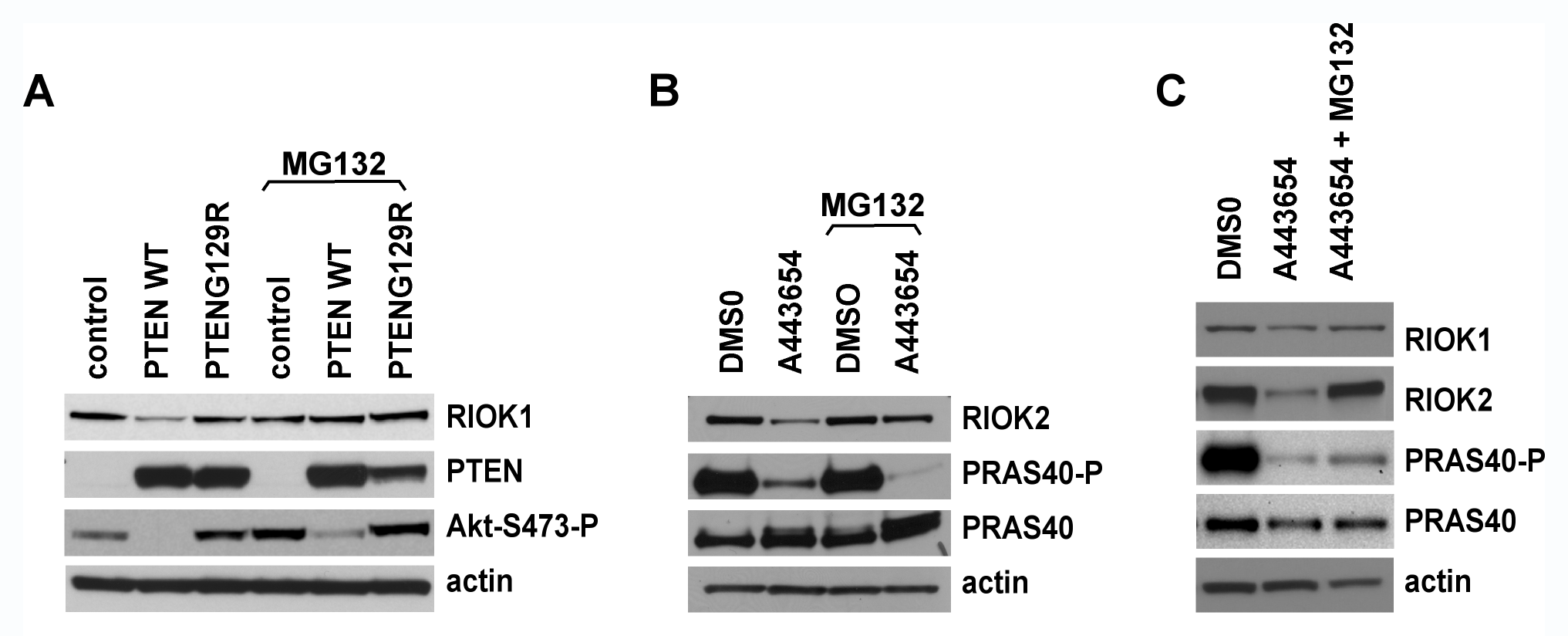

Supplement: Figure S7 — Akt signaling regulates RIO kinase protein stability. (A) U87MG-ΔEGFR cells were infected with retroviruses containing PTEN, PTEN-G129R (catalytically inactive), or empty vector. Cells were serum-starved for 48 hours and treated for 8 hours with 10 µM MG132, a proteosomal inhibitor. Reduced Akt phosphorylation at Serine-473 is evidence of inhibition by PTEN. (B, C) U87MG-ΔEGFR (B) cells or GBM301 (C) cells were treated with DMSO or 2 µM A443654 with and without 10 µM MG132 for 10 hrs. Akt inhibition is evidenced by reduced PRAS40 phosphorylation in A443664 treated samples. (TIF) [file pgen.1003253.s007.tif]

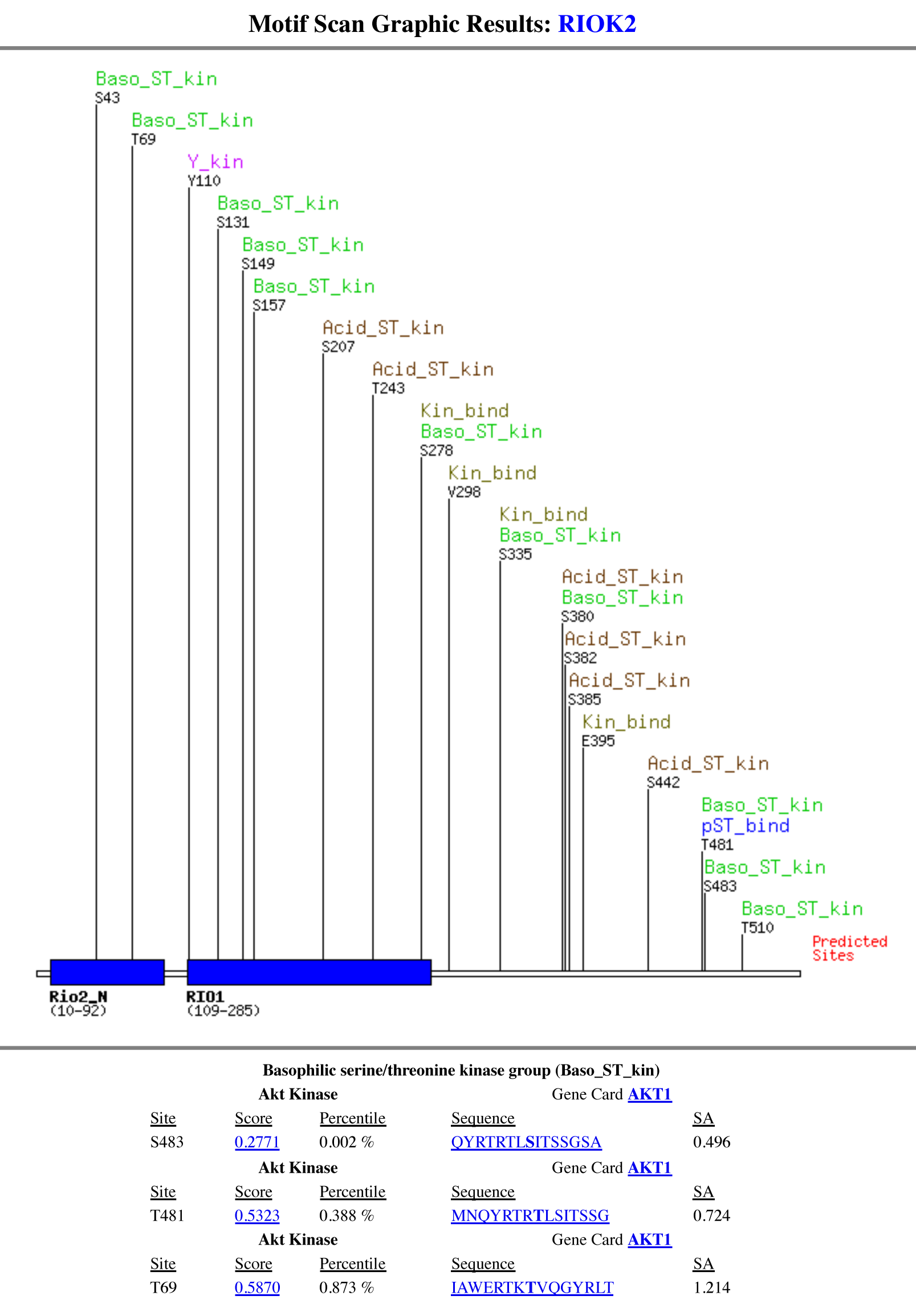

Supplement: Figure S8 — RIOK2 motif scan. The RIOK2 protein sequence was examined with the Scansite Motif Scanner (http://scansite.mit.edu/motifscan_seq.phtml) [75]. The kinase domain, which is highly similar to that of RIOK1 (RIO1), is indicated in blue. Potential phosphorylation sites are indicated by residue, and lower scores indicate that the predicted site falls into the top percentiles set by high stringency. Serine-483 in RIOK2 has been confirmed as a site of phosphorylation by several unpublished proteomic analyses available at PhosphoSitePlus (http://www.phosphosite.org) [76]. (TIF) [file pgen.1003253.s008.tif]

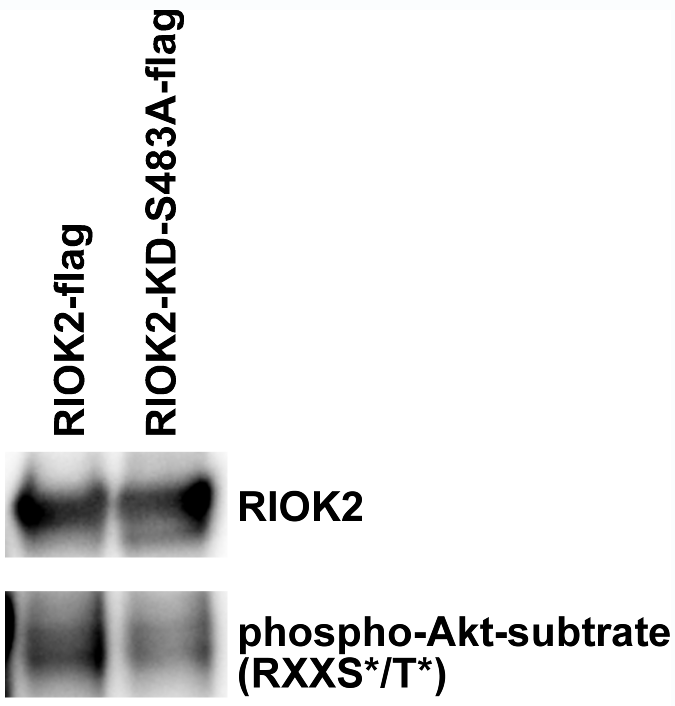

Supplement: Figure S9 — Potential phosphorylation of RIOK2 by Akt. Epitope-tagged RIOK2 (RIOK2-flag) was immunoprecipitated and detected by antibodies specific to Akt substrates phosphorylated on serine or threonine at characteristic Akt target sites (RXXS/T). RIOK2-KD-S483A-flag is an epitope tagged mutant form of RIOK2 which contains a serine-to-alanine change in Serine-483, which is a candidate Akt phosphorylation site (see Figure S8) and two point mutations that render RIOK2 kinase dead to block potential autophosphorylation [18]. Mutation of Serine-483 and the kinase domain did not block the ability of the Akt substrate antibody to detect RIOK2, indicating that other phosphorylation sites in RIOK2 are also recognized by the antibody. (TIF) [file pgen.1003253.s009.tif]

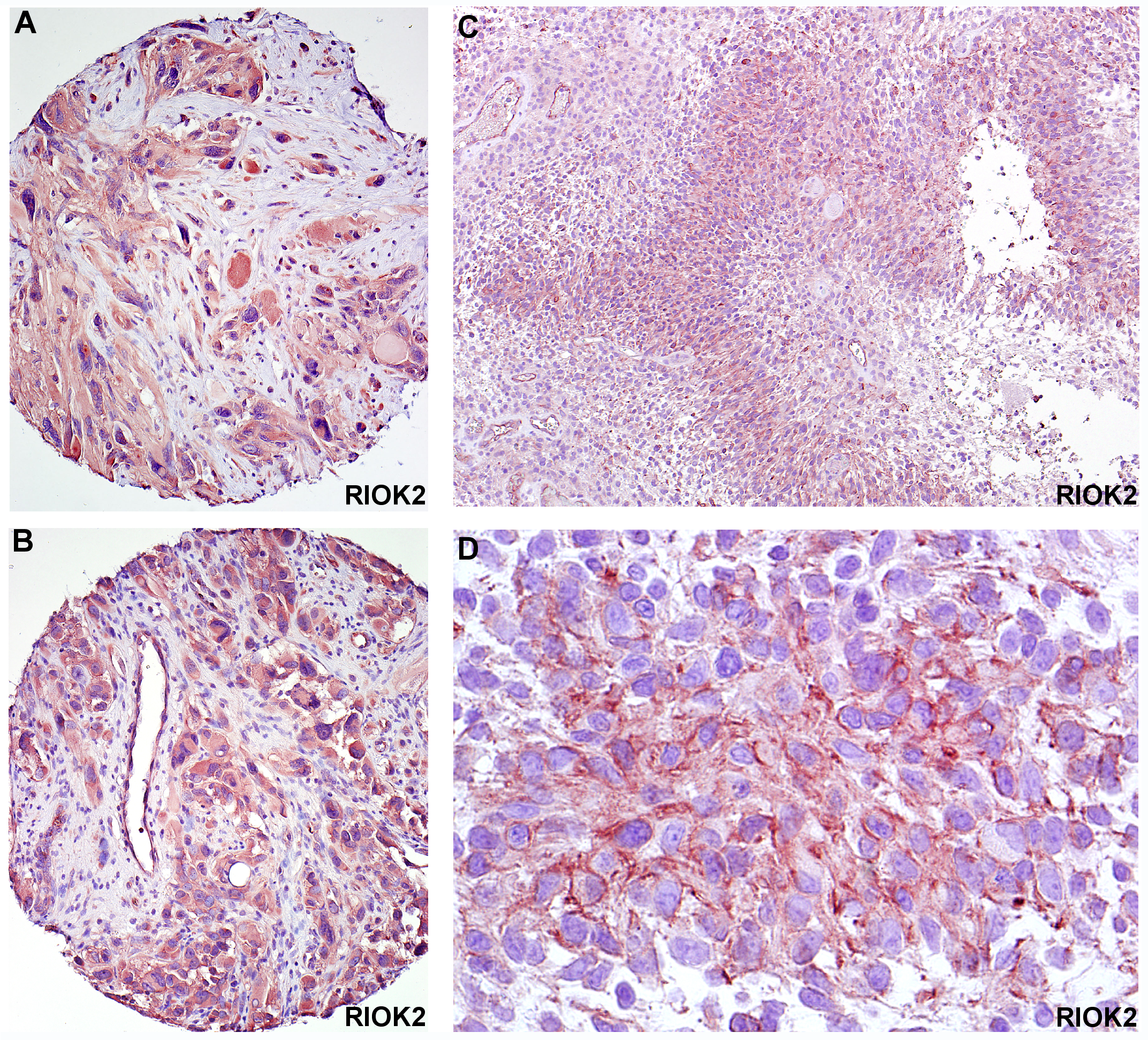

Supplement: Figure S10 — RIOK2 expression in the giant cell and pseudopallisade fractions of GBM tumors. Immunohistochemical staining for RIOK2 (reddish brown), with hematoxilin counterstain. Wider views of heterogeneous RIOK2 immunoreactivity in ΔEGFR-positive human GBMs with giant cell components (A, B), cropped section of A also shown in Figure 4B. Wider view showing RIOK2 immunoreactivity in pseudopallisades (C), also shown cropped in Figure 4D, with high magnification (D) to show enrichment for RIOK2 present in the cellular fraction of pseudopallisades. (TIF) [file pgen.1003253.s010.tif]

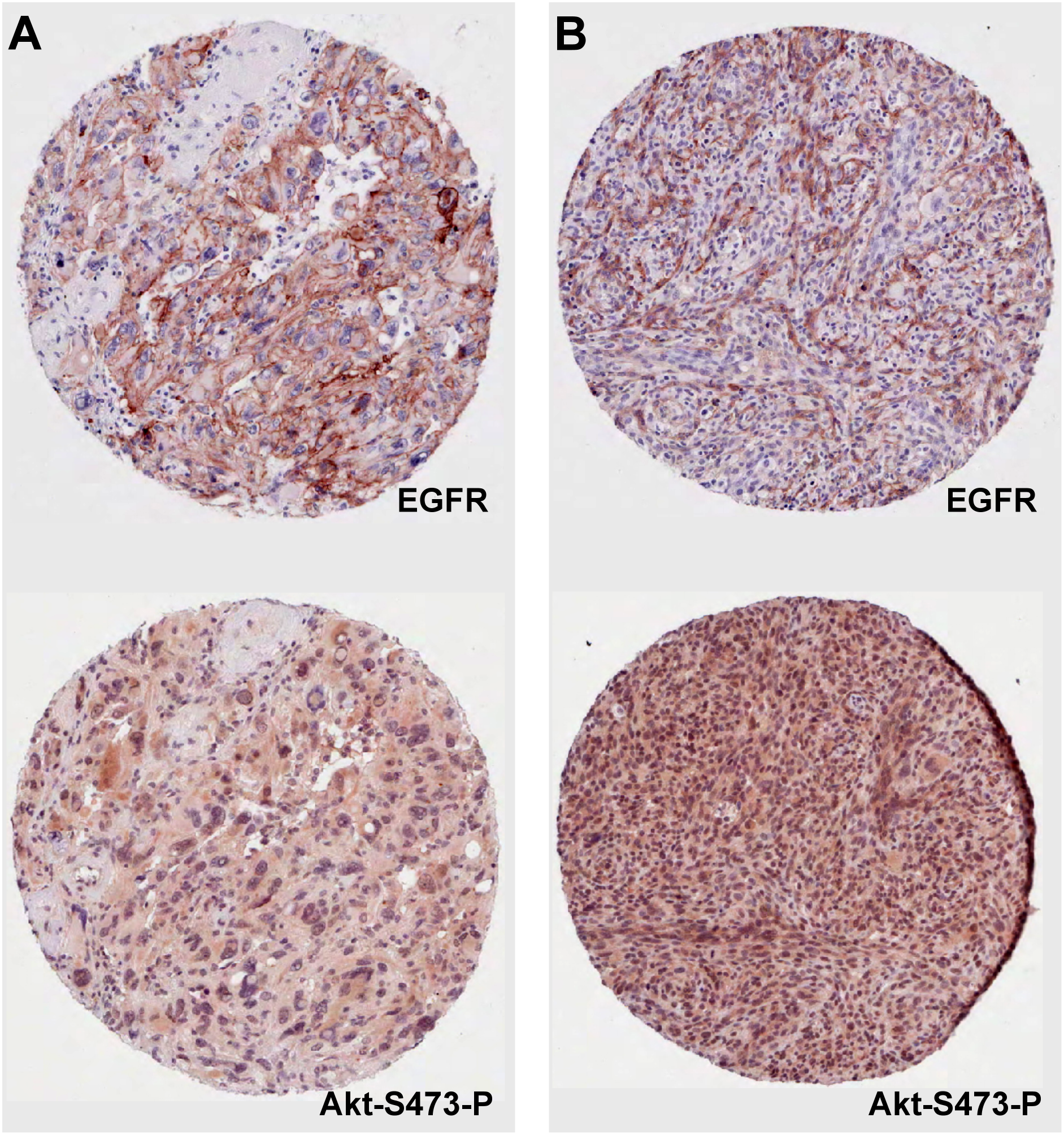

Supplement: Figure S11 — Akt and EGFR in GBM tumor tissues positive for RIOK2 expression. Immunohistochemical stains for EGFR and Akt done on sections from the same tumor samples stained for RIOK2 in Figure 4B/Figure S10A (A) and Figure 4E (B). Both tumors show strong expression of EGFR and phosphorylated Akt (Akt-S473-P). (TIF) [file pgen.1003253.s011.tif]

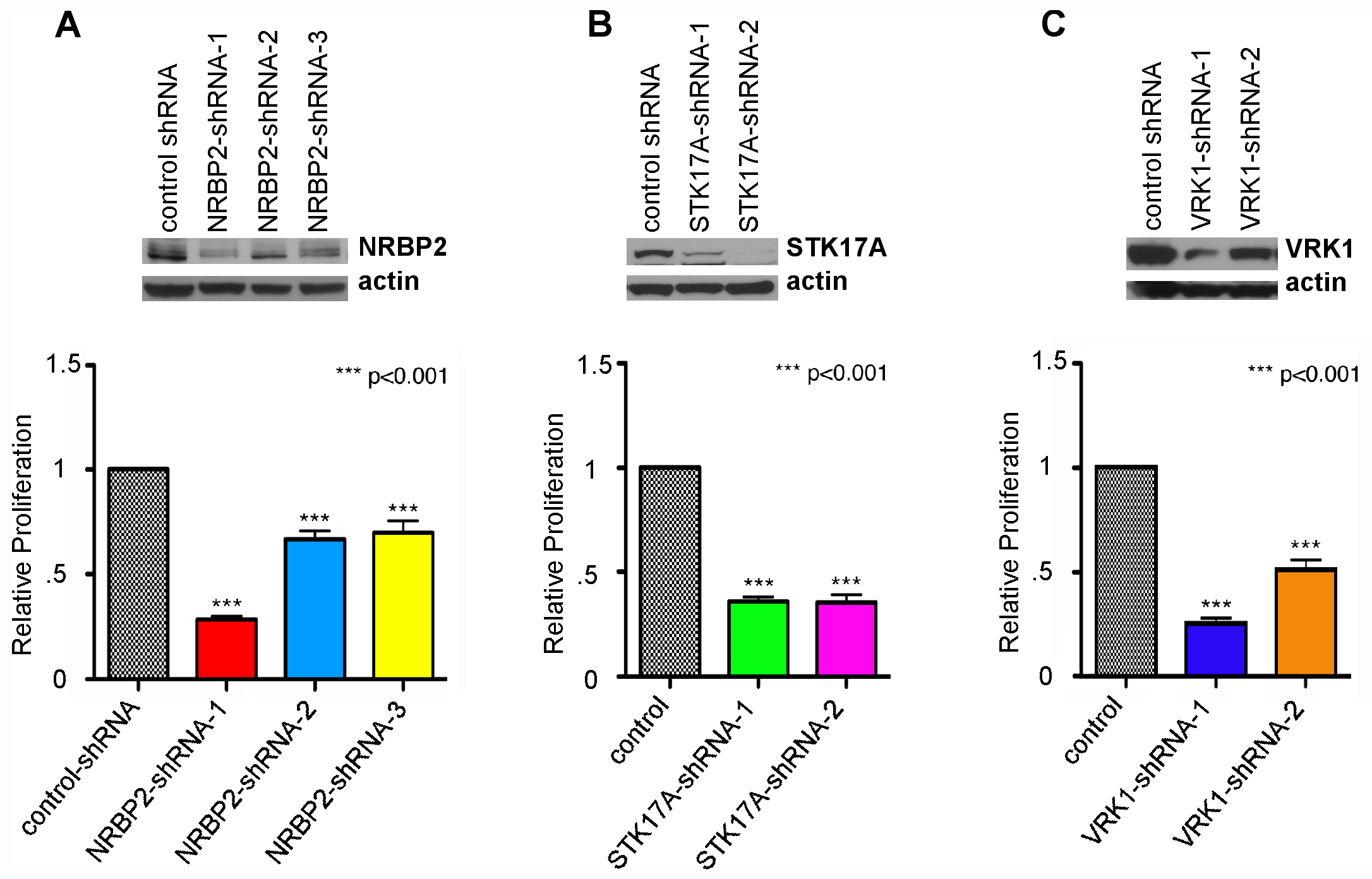

Supplement: Figure S12 — WST1 proliferation assays reveal a requirement for modifier kinases in GBM cells. (A–C) WST-1 assays performed on U87MG cells for indicated genes. Following selection for shRNA expression, proliferation was measured with WST-1 reagent and quantified as the fold increase in absorbance between day 0 and day 3, normalized to controls treated with a nontargeting shRNA. 2–3 shRNAs tested per kinase. P values refer to one-way ANOVA with Dunnett post test. (TIF) [file pgen.1003253.s012.tif]

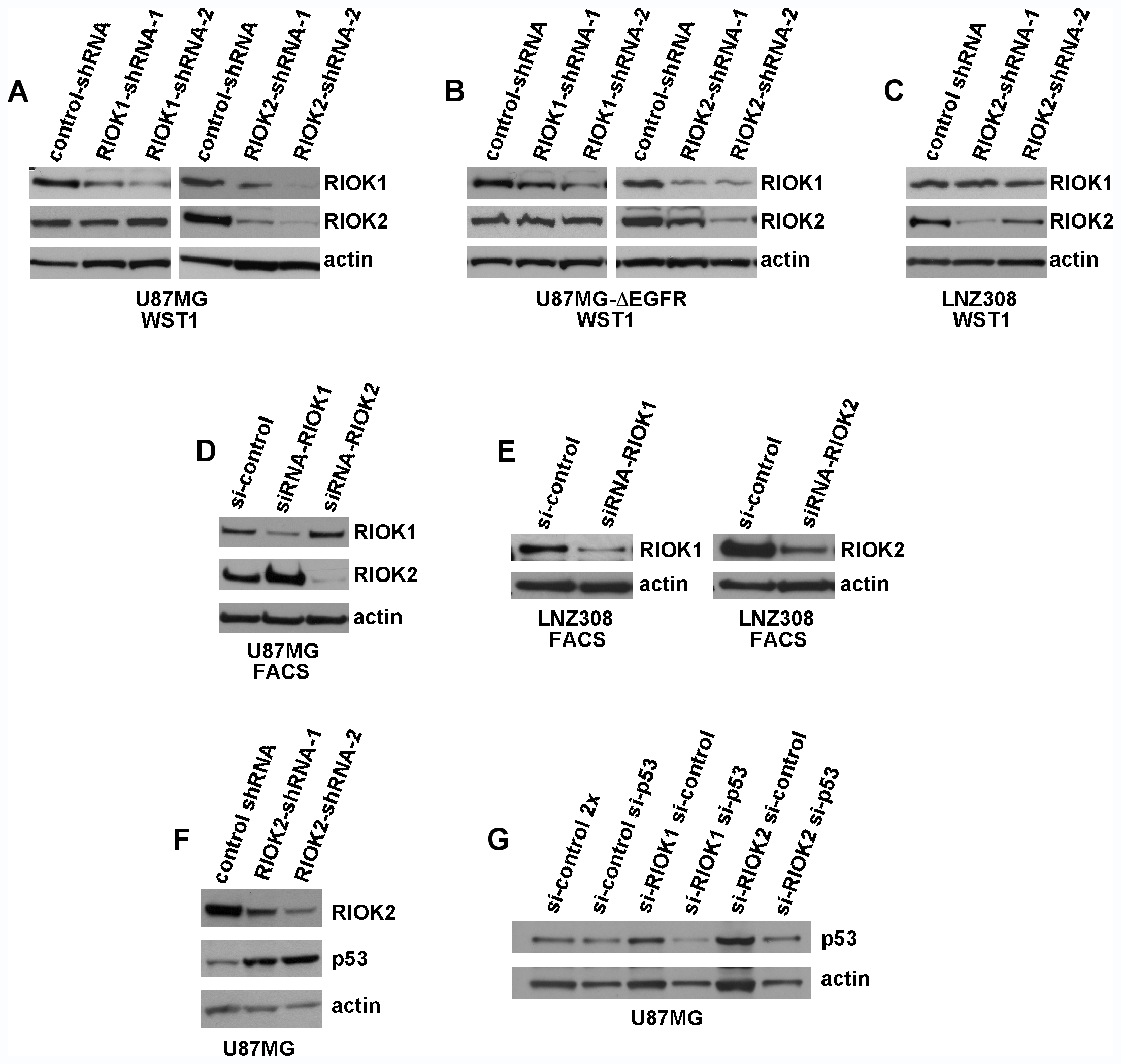

Supplement: Figure S13 — RIOK knockdown verification for Figure 5. (A, B) Verification of RIOK1 and RIOK2 knockdown in U87MG (A) and U87MG-ΔEGFR (B) cells treated with the indicated shRNAs and subjected to WST1 assays as shown in Figure 5A (left panels). (C) Verification of RIOK2 knockdown in LNZ308 (C) cells treated with the indicated shRNAs and subjected to WST1 assays as shown in Figure 5A (right panel). (D, E) Verification of RIOK1 and RIOK2 knockdown in U87MG (D) and LNZ308 (E) cells treated with the indicated siRNAs and subjected to FACS analysis for cell cycle progression as shown in Figure 5B. (F) Upregulation of p53 upon RIOK2 knockdown with shRNAs. (G) Verification of p53 knockdown in U87MG cells treated with siRNAs against p53 and the RIOKs and subject to FACS analysis of apoptosis as shown in Figure 5E. (TIF) [file pgen.1003253.s013.tif]

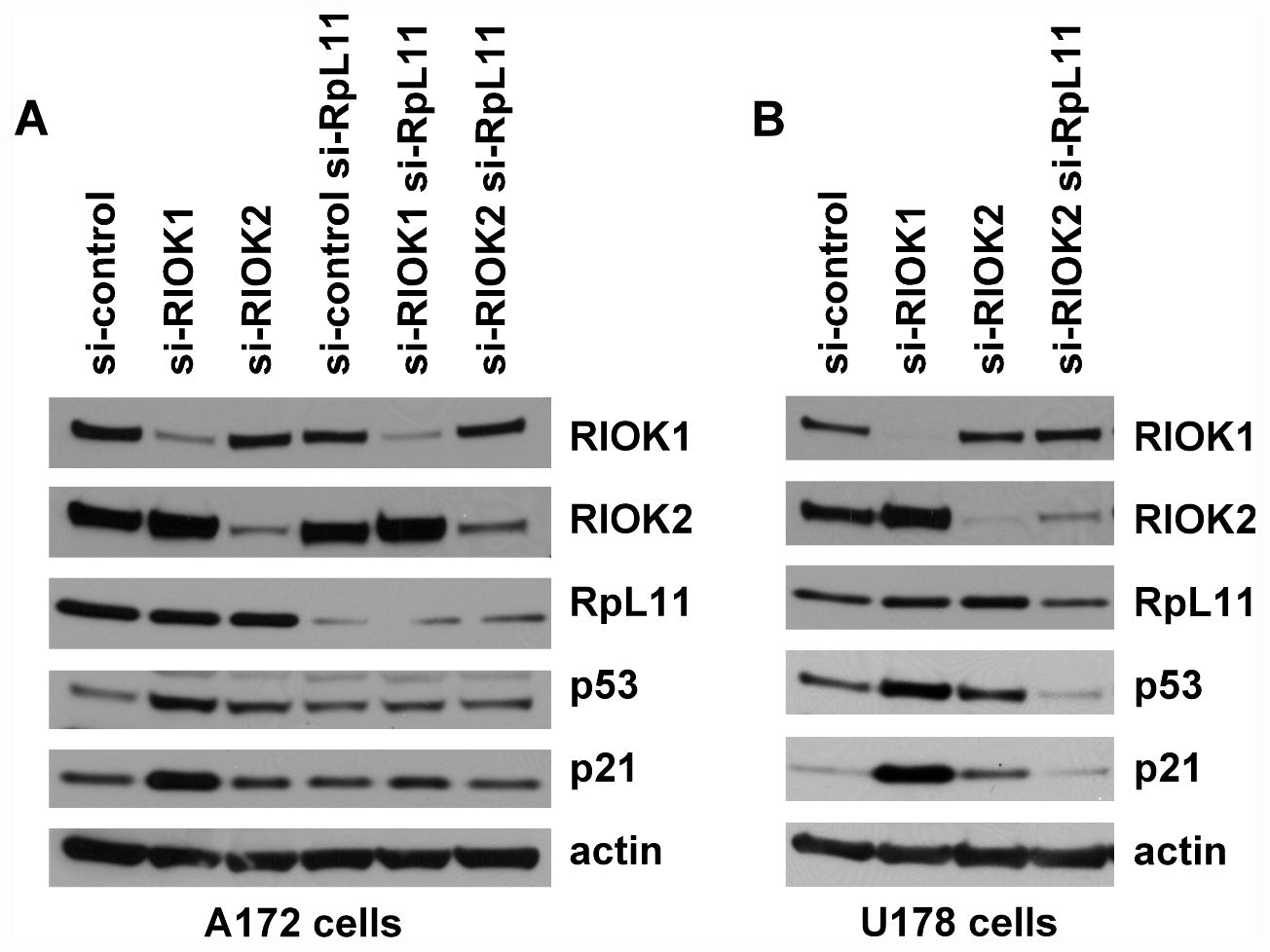

Supplement: Figure S14 — Loss of RIOK1 and RIOK2 induces L11-dependent p53 upregulation. RIOK1 or RIOK2 knockdown upregulates p53 and p21 expression in A712 (A) and U178 (B) GBM cells, which is blocked by concurrent RpL11 knockdown. A172 cells are PTEN-mutant and EGFR-mutant, U178 cells are PTEN-mutant, and both are wild-type for p53 [38], [39], [50]. For RpL11 co-knockdown experiments, cells were treated with equivalent amounts of siRNAs mixed 1∶1 for all control and experimental samples. Cells were harvested 72 hrs post-transfection with siRNAs. (TIF) [file pgen.1003253.s014.tif]

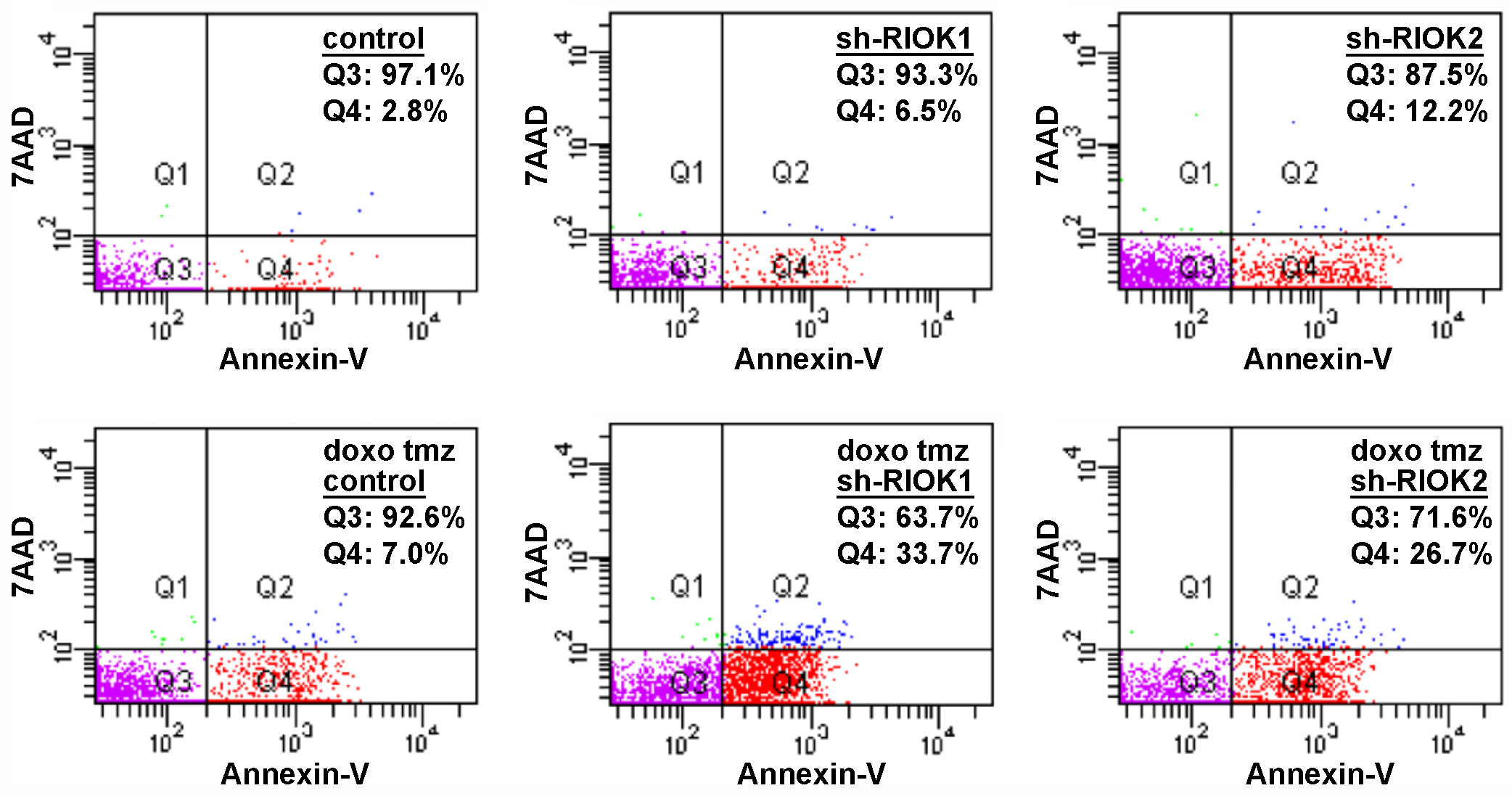

Supplement: Figure S15 — RIOK loss chemosensitizes GBM cells. Representative scatter plots of FACS-based quantification of chemosensitivity. U87MG cells were treated with 1 µg/mL doxorubicin and 100 µM temozolomide for 12 hrs beginning 96 hrs post shRNA infection. Treated cells were collected and stained live for 7AAD and Annexin-V. 7AAD alone identifies dead cells (Q1). Annexin V identifies cells in early apoptosis when alone (Q4) and in late stage apoptosis when coincident with 7AAD (Q2). Viable cells (Q3) stained for neither. The percentage of Annexin-V-positive cells present upon doxorubicin and temozolomide treatment significantly increased in RIOK1-shRNA and RIOK2-shRNA treated cells compared to cells treated with a non-targeting control shRNA. (TIF) [file pgen.1003253.s015.tif]

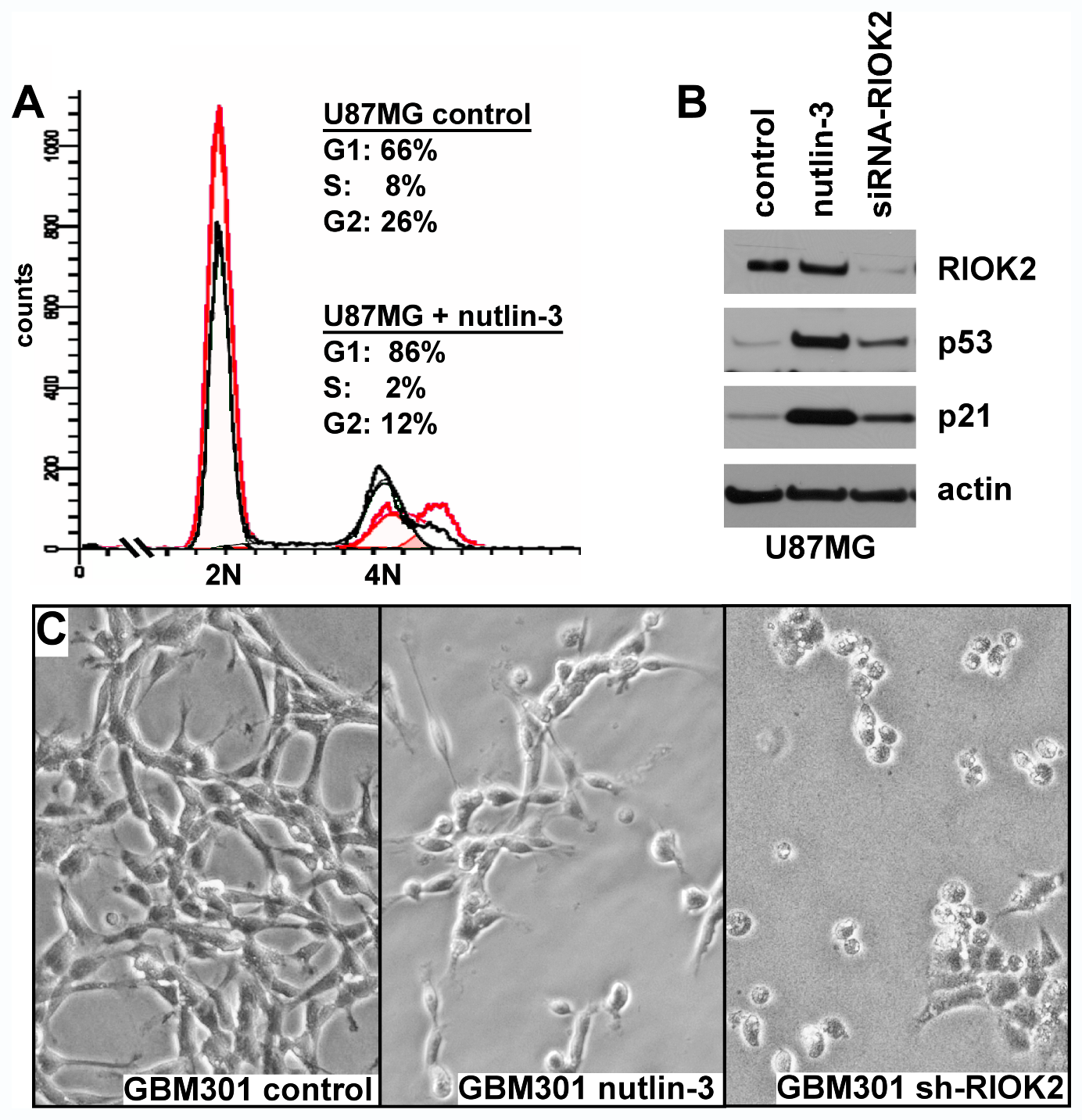

Supplement: Figure S16 — Nutlin-3 treatment does not phenocopy RIOK2 loss. (A) U87MG cells treated with 10 µM nutlin-3 (red) or DMSO (control, black) for 48 hrs prior to fixation and propidium iodide staining for DNA content for cell cycle analysis by FACS. (B) Immunnoblot showing that nutlin-3 treatment significantly increased p53 and p21 levels in U87MG cells, as compared to both control cells and siRNA-RIOK2 treated cells. (C) GBM301 cells plated adherently and infected with a GFP control lentivirus (left panel) or an shRNA lentivirus targeting RIOK2 (right panel). Brightfield images taken 96 hrs post-infection. RIOK2 protein levels drop starting about 72 hrs post-infection, such that 96 hrs is equivalent to 24 hrs of knockdown. Adherent GBM301 cells treated with 10 µM nutlin-3 for 24 hrs (middle panel). Nutlin-3 largely decreased growth of GBM301 cells, whereas RIOK2 knockdown more prominently stimulated apoptosis, yielding many pyknotic and vacuolated cells. (TIF) [file pgen.1003253.s016.tif]

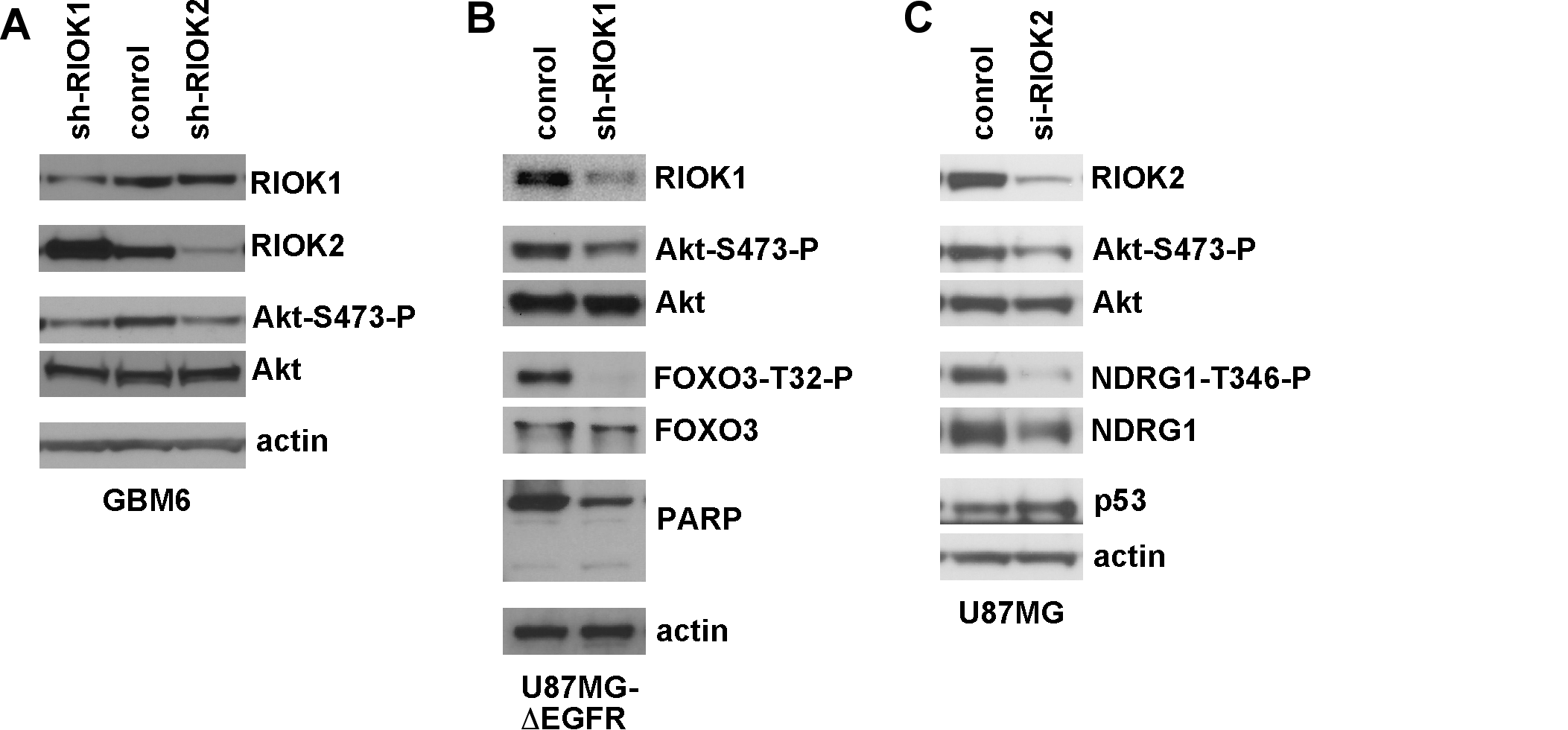

Supplement: Figure S17 — Loss of RIOK1 and RIOK2 function reduces TORC2-Akt signaling. (A) GBM6, a ΔEGFR-positive and p53 mutant neurosphere line. RIOK1 or RIOK2 knockdown caused reduced phosphorylation of Akt on the TORC2 target site, Serine-473, which is clear relative to total Akt protein. Cells harvested 5 days post-infection with viral vectors that target RIOK1/2 or a control vector, treated with 25 µM ZVAD for 48 hrs prior to harvest. (B) RIOK1 knockdown caused reduced phosphorylation of Akt on Serine-473 in U87MG-ΔEGFR cells. Reduced phosphorylation of FOXO3 at the TORC2-dependent Akt target site was detected upon RIOK1 knockdown. Cells harvested 5 days post-infection with viral vectors that target RIOK1/2 or a control vector, treated with 25 µM ZVAD for 48 hrs prior to harvest. (C) RIOK2 knockdown caused reduced phosphorylation of Akt on Serine-473 in U87MG cells. RIOK2 knockdown was also associated with reduced phosphorylation of NDRG1, aTORC2 read-out, and increased p53 protein levels. Cells treated with nontargeting control siRNAs or RIOK2 siRNAs, harvested 96 hrs post-transfection. (TIF) [file pgen.1003253.s017.tif]

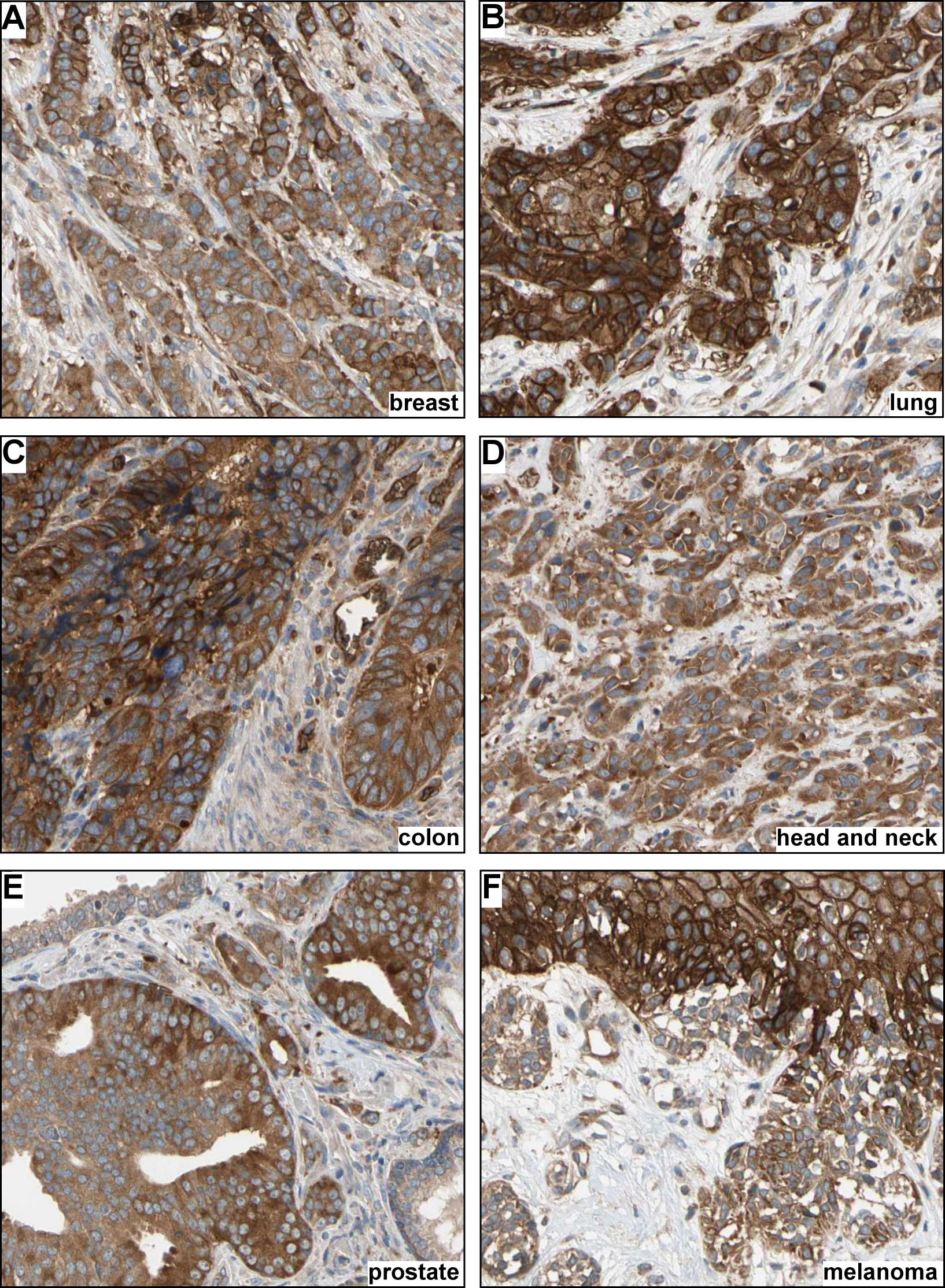

Supplement: Figure S18 — Expression of RIOK2 in other tumor types. (A–F) Representative immunohistochemical stains for RIOK2 performed on malignant tumor tissue, all done as part of the Human Protein Atlas Project [73]. (A) breast cancer (duct carcinoma), (B) lung cancer (squamous cell carcinoma), (C) colorectal cancer (adenocarcinoma), (D) head and neck cancer (squamous cell carcinoma), (E) prostate cancer (adenocarcinoma), and (F) malignant melanoma. RIOK2 showed strong expression in 92–100% of colon and head and neck tumors examined, in 50–58% of lung, prostate, and melanoma tumors examined, and in 25% of breast tumors examined (complete analysis and images available at www.proteinatlas.org). (TIF) [file pgen.1003253.s018.tif]
