# Supplementary material for: A Kinome-Wide RNAi Screen in Drosophila Glia Reveals That the RIO Kinases Mediate Cell Proliferation and Survival through TORC2-Akt Signaling in Glioblastoma
Source: PLoS Genet. 2013 Feb 14;9(2):e1003253. doi: 10.1371/journal.pgen.1003253 (PMC3573097; doi:10.1371/journal.pgen.1003253)
Supplement: Text S1 — Supplemental Results and Materials and Methods. Additional detail on validation of novel kinases in mammalian systems, screening methodology, and shRNA and siRNA sequences. (DOC) [file pgen.1003253.s029.doc]

**Text S1 Supplemental Results and Materials and Methods for**

**A kinome-wide RNAi screen in *Drosophila* glia reveals that the RIO kinases mediate cell proliferation and survival through TORC2-Akt signaling in glioblastoma**

Read, Renee D.^1, 6^*, Tim R. Fenton^2^, German G. Gomez^2^, Jill Wykosky^2^, Scott R. Vandenberg^3^, Ivan Babic^2^, Akio Iwanami^4^, Huijun Yang^2^, Webster K. Cavenee^2, 5^, Paul S. Mischel^2,3^, Frank B. Furnari^2,3^, and John B. Thomas**^1^**.

1) Molecular Neurobiology Laboratory, The Salk Institute for Biological Studies, 10010 North Torrey Pines Rd, La Jolla, CA

2) Ludwig Institute for Cancer Research, University of California at San Diego, La Jolla, CA

3) Department of Pathology, School of Medicine, University of California at San Diego, La Jolla, CA

4) Department of Orthopaedic Surgery, Keio University School of Medicine, Tokyo, Japan

5) Department of Medicine, School of Medicine, University of California at San Diego, La Jolla, CA

6) current address: Department of Pharmacology,

Emory University School of Medicine, Atlanta, GA

*corresponding author: renee.read@emory.edu

**Inventory of Text S1**

Supplemental Results p. 3-5

Supplemental Materials and Methods p. 6-7

Supplemental References p. 8

**Supplemental Results**

Kinases identified in our *Drosophila* screen may represent human kinases directly involved in GBM pathogenesis. Novel kinases that suppress fly glial neoplasia when their expression is reduced are of particular interest because their human orthologs may be promising new targets for therapeutic inhibition. Rapid translation of our findings into human GBM models could lead to the discovery of new pathways that govern glial tumorigenesis in mammalian systems. There are 27 human orthologs for the 16 novel *Drosophila* modifier kinases (Table S8). Thus, we assessed these human kinases for involvement in GBM. This was accomplished by analyzing their expression, mutation, and/or function in GBM tumors and cell lines.

Microarray gene expression data from The Cancer Genome Atlas (TCGA, http://tcga-portal.nci.nih.gov/tcga-portal/AnomalySearch.jsp) for over 400 GBM tumors shows that, of the 22 kinases detected, transcripts for two kinases are upregulated in EGFR-overexpressing tumors relative to normal control tissue (Table S9) [1]. Both kinases that show upregulation, STK17A and STK17B, are orthologs of *Drosophila* Drak, a suppressor. For 19 kinases, gene expression is comparable between tumor and normal tissue, suggesting that the majority of modifier orthologs are not dysregulated in GBMs at the mRNA level.

Examination of tumor-specific copy number alterations catalogued by the TCGA revealed that no modifier orthologs showed significant deletion, while one, STK17A, showed significant copy gain (>3% of tumors tested). STK17A maps to chromosome 7 near EGFR, and is subject to copy gain in 28% (104/372) of tumors, respectively, nearly all of which show EGFR amplification. STK17A may become amplified because it contributes to tumorigenesis, or, alternately, STK17A may become amplified because it maps closely to EGFR.

The TCGA and Sanger Center uncovered low penetrance point mutations in the modifier orthologs TNK2 (3 /151 patients), TAOK3 (1/159 patients), NRBP2, PRKG1, and TLK1 (1/9 patients tested each) [1,2]; no other orthologs have been systematically sequenced to date. The relevance of these mutations to gliomagenesis awaits experimental analysis.

Since many kinases are regulated post-translationally, we expected to find tumor-specific alterations in protein expression. To assess this, we queried the Human Protein Atlas ([www.proteinatlas.org](http://www.proteinatlas.org/), HPA), a publically available immunohistochemical (IHC) database on protein expression in normal and cancerous human tissues [3]. HPA IHC data revealed that 3 kinases show strong-moderate protein expression in at least 30% of high-grade malignant gliomas and GBM cell lines, 3 kinases show moderate expression in less than 30% of glioma biopsies, and another 3 kinases show weak-undetectable protein expression (Table S10, Figure S3). HPA IHC data showed that one of the strongly expressed kinases - RIOK2 - is expressed at low-undetectable levels in normal neurons in control brain tissue specimens (Table S10), which implies that RIOK2 protein may be specifically upregulated in tumors.

Thus, publically available tumor genomic and proteomic databases provided suggestive evidence that at least 8 modifier orthologs are subject to alterations in GBMs. Taking into account these data, bioinformatic gene annotation, and available reagents, we selected 9 kinases (noted on Table S8) for examination of protein expression in cultured GBM cells in order to delimit candidates for functional analysis. To discern if any of these 9 kinases become expressed in the context of oncogenic EGFR signaling and/or PTEN mutation, we determined which kinases were expressed in U87MG GBM cells, which are null mutant for PTEN, and in U87MG cells engineered to express ΔEGFR at levels commonly detected in primary tumors [4]. All 9 kinases were expressed in U87MG cells (Figure S4). Protein levels of 2 kinases – RIOK1, RIOK2 - were upregulated in the presence of ΔEGFR (Figure 2A, Figure S4). Further examination of RIOK1 and RIOK2 expression and function in GBM cells and tumor tissues is presented in the main text.

Coincident with our expression studies, we began functional studies of orthologs of modifier kinases that yielded strong growth suppression of both dEGFRλ;dp110^CAAX^ and ΔEGFR mutant glia in *Drosophila*, with an emphasis on orthologs subject to alterations in GBM tumors. To determine if orthologs of modifier kinases are required for survival and/or proliferation of GBM cells, we performed WST1 assays on cultured U87MG cells treated with shRNA constructs that knockdown individual kinases of interest (Figure 5A, Figure S12, data not shown). In addition to showing that RIOK1 and RIOK2 were required for GBM cell proliferation and survival (see main text for results of RIOK1 and RIOK2 experiments), results from our WST1 assays suggested that NRBP2, STK17A, and VRK1 are also required in survival and/or proliferation of GBM cells (Figure S12). To date, these kinases have been not implicated in GBM, and have not been previously studied in other cancers. Yet, these kinases have connections to biological processes implicated in gliomagenesis; NRBP2 is highly expressed in neural stem cells; STK17A is homologous to the *Drosophila* kinase Drak, which regulates cytoskeletal remodeling and organ growth; and mutations in VRK1, which phosphorylates p53, are associated with neurodevelopmental defects and stem cell failure [5,6,7,8,9]. Continued studies of these kinases as well as the RIO kinases may reveal new mechanisms and fundamental cellular defects underlying gliomagenesis.

**Supplementary Materials and Methods**

**Fly stocks and genetics**

The screen was based on a one-step cross that generated progeny containing a single RNAi construct exclusively expressed in GFP-labeled glia along with dEGFRλ and dp110^CAAX^. Transgenes were overexpressed using *repo-Gal4* transcriptional driver, which is expressed in almost all post-embryonic glial cell types. The Gal80 transcriptional repressor was used to block *repo-Gal4* transactivation of GFP, dEGFR*λ*, and dp110^CAAX^ transgenes in the parental generation. In the F1 generation, Gal80 segregated away from *repo-Gal4* to allow glial-specific expression of all transgenes, including genetically encoded RNAi constructs. Screening was performed using fluorescence microscopy to visualize GFP-labeled glia in living larvae, and phenotypes were confirmed with confocal microscopy. Each positive-scoring RNAi construct was tested at least twice, and RNAi constructs with predicted off-target activity towards known modifiers were excluded.

Secondary screens in wild-type cells were based on one-step crosses of positive scoring dsRNA constructs to cell-type specific Gal4- drivers for glia (*repo-Gal4*), neurons (*elav-Gal4*), neuroblasts (*wor-Gal4*), and epithelial progenitor cells in the eye (*ey-Gal4*). For glia, gross glial phenotypes were observed in larvae: 3^rd^ instar larval brains were fixed, stained for glial cell numbers (anti-Repo for glial cell nuclei), labeled genetically with GFP for glial cell morphology, and imaged as described [10]. Gross larval phenotypes were recorded, and whole animal viability through to adulthood was monitored. For epithelial progenitor cells, adult eye phenotypes were observed, which is a reliable measure of developmental proliferation and apoptosis. For neuroblasts, our results were compared to published tests of many RNAi constructs with another neuroblast-specific Gal4-driver (*insc-Gal4*) [11].

For EGFR inhibitor treatments, gefitinib was mixed into *Drosophila* media to a final concentration of 300 or 500 µM. For treatment, age-matched hatched 1^st^ instar larvae were placed on gefitinib-containing media and reared for ~96 hrs prior to scoring/imaging, or until adulthood to monitor rescue.

**Mammalian tissue culture and RNAi**

RIOK1 and RIOK2 siRNA target sequences (Dharmacon) listed below:

RIOK1 siGenome pool: GCGCCAACGUCAAUGAUUU, GAGCAUAUAUUCCUAGAAC, GGAAAGGACUUGUUCUGAU, GAAAUAGCAUCUCAAAGGA

RIOK1 On-Target Plus pool: CCAAUAAUGCUAAGAAGUC, GGAGGCGUGUAUAUCAUUG, GAACAUGGAUGCUUAUCUC, GCGCCAACGUCAAUGAUUU

RIOK2 siGenome pool: GAAGAACCAUGAAAUUGUU, GGACAACAAUUUGCAUUAA, GAACGGAACUGUCUAGAAG, GGAAGAACCUCGUUUCGAA

RIOK2: On-Target Plus pool: UGGGAGCUAUGAAUCAGUA, GUCCAGGGCUAUCGGUUGA, CCAGAUGGGUGUUGGCAAA, UGAAGGAAUUUGCCUAUAU

RpL11 siGenome pool: GAACUUCGCAUCCGCAAAC, UAAAGGUGCGGGAGUAUGA, AAAGCUAGAUACACUGUCA, AAAGAUUGCUGUCCACUGC

**References**

1. McLendon R, Friedman A, Bigner D, Van Meir EG, Brat DJ, et al. (2008) Comprehensive genomic characterization defines human glioblastoma genes and core pathways. Nature 455: 1061-1068.

2. Hunter C, Smith R, Cahill DP, Stephens P, Stevens C, et al. (2006) A hypermutation phenotype and somatic MSH6 mutations in recurrent human malignant gliomas after alkylator chemotherapy. Cancer Res 66: 3987-3991.

3. Uhlen M, Oksvold P, Fagerberg L, Lundberg E, Jonasson K, et al. (2010) Towards a knowledge-based Human Protein Atlas. Nat Biotechnol 28: 1248-1250.

4. Huang HS, Nagane M, Klingbeil CK, Lin H, Nishikawa R, et al. (1997) The enhanced tumorigenic activity of a mutant epidermal growth factor receptor common in human cancers is mediated by threshold levels of constitutive tyrosine phosphorylation and unattenuated signaling. J Biol Chem 272: 2927-2935.

5. Neubueser D, Hipfner DR (2010) Overlapping roles of Drosophila Drak and Rok kinases in epithelial tissue morphogenesis. Mol Biol Cell 21: 2869-2879.

6. Larsson J, Forsberg M, Brannvall K, Zhang XQ, Enarsson M, et al. (2008) Nuclear receptor binding protein 2 is induced during neural progenitor differentiation and affects cell survival. Mol Cell Neurosci 39: 32-39.

7. Renbaum P, Kellerman E, Jaron R, Geiger D, Segel R, et al. (2009) Spinal muscular atrophy with pontocerebellar hypoplasia is caused by a mutation in the VRK1 gene. Am J Hum Genet 85: 281-289.

8. Lopez-Borges S, Lazo PA (2000) The human vaccinia-related kinase 1 (VRK1) phosphorylates threonine-18 within the mdm-2 binding site of the p53 tumour suppressor protein. Oncogene 19: 3656-3664.

9. Choi YH, Park CH, Kim W, Ling H, Kang A, et al. (2010) Vaccinia-related kinase 1 is required for the maintenance of undifferentiated spermatogonia in mouse male germ cells. PLoS One 5: e15254.

10. Read RD, Cavenee WK, Furnari FB, Thomas JB (2009) A drosophila model for EGFR-Ras and PI3K-dependent human glioma. PLoS Genet 5: e1000374.

11. Neumuller RA, Richter C, Fischer A, Novatchkova M, Neumuller KG, et al. (2011) Genome-Wide Analysis of Self-Renewal in Drosophila Neural Stem Cells by Transgenic RNAi. Cell Stem Cell 8: 580-593.
